# Modified EASIX score on day 7 predicts survival and non-relapse mortality in pediatric acute leukemia undergoing haploidentical stem cell transplantation

**DOI:** 10.3389/fimmu.2025.1646640

**Published:** 2026-01-12

**Authors:** Kai Cui, Senlin Zhang, Yueke Du, Qingwei Wang, Yutan Chai, Li Gao, Yixin Hu, Bohan Li, Yuanyuan Tian, Yongping Zhang, Shuiyan Wu, Shaoyan Hu, Jie Li

**Affiliations:** 1 Department of Hematology and Oncology, Children’s Hospital of Soochow University, Suzhou, China; 2 Pediatric Intensive Care Unit, Children’s Hospital of Soochow University, Suzhou, China; 3 Jiangsu Pediatric Hematology and Oncology Center, Suzhou, China

**Keywords:** clinical outcomes, haploidentical, hematopoietic stem cell transplantation, m-EASIX, pediatric

## Abstract

**Background:**

The Endothelial Activation and Stress Index (EASIX) has been validated in adult hematopoietic stem cell transplantation (HSCT) recipients as a predictor of overall survival (OS), non-relapse mortality (NRM), and endothelial-related complications. However, the prognostic significance in children receiving haploidentical donor (HID) transplantation based on myeloablative conditioning (MAC) is still uncertain.

**Method:**

Pediatric leukemia patients who underwent HID transplantation at the Children’s Hospital of Soochow University between January 2020 and December 2024 were retrospectively reviewed. Based on transplantation dates, patients were assigned to training and validation cohorts. EASIX (lactate dehydrogenase (U/L) × creatinine (mg/dL)/platelet (10^9^ cells/L), sEASIX (excluding creatinine), and m-EASIX (substitutes creatinine with C-reactive protein (mg/dL) were calculated at pre-conditioning, day 0, day 7, day 14, and day 30. All indices were log2-transformed investigate their relevance to clinical outcomes.

**Results:**

In the training cohort, we stratified patients into groups with high or low D7-m-EASIX expressions, based on the optimal cutoff of 4.1 identified using maximally selected log-rank statistics. In the training cohort, D7-m-EASIX >4.1 was an independent predictor of overall survival (OS, HR:2.35, P = 0.013), relapse-free survival (RFS, HR:1.85, P = 0.047), NRM (HR:3.32, P = 0.009), and was associated with II-IV acute graft-versus-host disease (aGVHD, HR:2.16, P = 0.003) in multivariate analysis. The validation cohort supported these results (OS [HR:6.56, P = 0.005], RFS [HR:3.20, P = 0.030], NRM [HR:5.32, P = 0.040], and II-IV aGVHD [HR:2.57, P = 0.008]).

**Conclusion:**

D7-m-EASIX is a simple and valuable prognostic biomarker for pediatric leukemia patients undergoing HID transplantation based on MAC.

## Introduction

Allogeneic hematopoietic stem cell transplantation (allo-HSCT) offers an effective curative strategy for acute leukemia. However, high non-relapse survival rate (NRM) following transplantation continues to pose a major clinical challenge. Conditioning regimens, immunosuppressor and alloreactivity can lead to endothelial injury, contributing to several post-transplant complications, such as graft-versus-host disease (GVHD), sinusoidal obstruction syndrome (SOS), and transplantation-associated microangiopathy (TMA), which are among the leading causes of NRM (1–3). Thus, it is crucial for early identification of endothelial injury to improve prognosis.

The endothelial activation and stress index (EASIX), formulated from three standard laboratory indicators, lactate dehydrogenase (LDH), creatinine, and platelet count (PLT), functions as a reliable marker for assessing endothelial injury severity (4). EASIX has been validated as a predictor of overall survival (OS), NRM and endothelial injury-related complications (4–8). However, considering the age-specific normal values for LDH and creatinine, the EASIX score has not been well validated in pediatric cohorts (4, 9).

Following the incorporation of antithymocyte globulin (ATG) -containing regimens and post-transplant cyclophosphamide into clinical practice, HID transplantation offers a practical alternative for individuals lacking a human leucocyte antigen-matched sibling donor (10). Relative to matched sibling donor transplantation, HID transplantation has been linked to a delay in platelet reconstitution and a greater overall incidence of both acute (aGVHD) and chronic GVHD (cGVHD) (11). To date, data on the utility of the EASIX score in exclusively HID cohorts remains limited.

Given that C-reactive protein (CRP) is a commonly used biomarker in hematologic malignancies (12). We evaluated the predictive utility of the original EASIX score along with two variants: the simplified EASIX (s-EASIX, omits creatinine) and the modified EASIX (m-EASIX, substitutes creatinine with CRP). Notably, a previous study has reported that the m-EASIX can predict severe cytokine release syndrome and immune effector cell–associated neurotoxicity syndrome following chimeric antigen receptor T-cell therapy (13).

## Method

### Patients

Patients with acute leukemia underwent HID transplantation at the Children’s Hospital of Soochow University between January 2020 and December 2024 were included. Patients from 2020 to 2022 formed the training cohort (n = 195), while those from 2023 to 2024 constituted the validation cohort (n = 109). Inclusion Criteria: 1. Diagnosed with acute leukemia or myelodysplastic syndromes; 2. Underwent first allo-HSCT; 3. Age <18 years at the time of HSCT; 4. Availability of complete laboratory data, including LDH, PLT, creatinine, and CRP.

Exclusion Criteria: 1. Age ≥18 years at the time of HSCT; 2. Prior chimeric antigen receptor T-cell therapy; 3. Receipt of autologous hematopoietic stem cell transplantation, cord blood transplantation, or a second transplantation; 4. Receipt of T-cell-depleted transplantation.5. Patients lacking key clinical or laboratory data were excluded from the study.

### Procedure

Myeloablative conditioning (MAC) regimens, incorporating either total body irradiation (4 Gy/day, days -7 to -5) or busulfan (3.2 mg/kg, days -7 to -4), were applied to all included patients. Cyclophosphamide (60 mg/kg for 2 days) combined with ATG (2.5 mg/kg from days -5 to -2) was administered. Beginning on day +6, granulocyte colony-stimulating factor (5 μg/kg/day) was provided and continued until the absolute neutrophil count surpassed 1 × 10^9^/L.

To prevent GVHD, all patients received mycophenolate mofetil (20–30 mg/kg/day from day −1 to +30, with the dose halved for 15 days afterward) and methotrexate (15 mg/m² on day +1, and 10 mg/m² on days +3, +6, and +11). In addition, either cyclosporine (target blood level: 200–250 ng/mL) or tacrolimus (target blood level: 10–15 ng/mL) was administered as part of the prophylactic regimen.

### EASIX, s-EASIX and m-EASIX

The formula for calculating the EASIX score is: LDH (U/L) × creatinine (mg/dL)/PLT (10^9^ cells/L). For s-EASIX and m-EASIX, the formulas were LDH (U/L)/PLT (10^9^ cells/L) and LDH (U/L) × CRP (mg/dL)/PLT (10^9^ cells/L), respectively. To reduce skewness, log_2_ normalization was applied to three formulas. Data were collected at predefined time points: pre-conditioning, day 0, day 7, day 14, and day 30. Patients who died prior to a given measurement point were analyzed based on data collected up to that point.

Serum CRP levels were measured using a commercial ELISA kit according to the manufacturer’s instructions (Elabscience Biotechnology Co., Ltd, Wuhan, China). The normal reference range was 0–8 mg/L. For calculation of m-EASIX, CRP values were converted from mg/L to mg/dL to match the formula.

### Definition

This study focuses on evaluating the association of EASIX, s-EASIX, and m-EASIX with NRM. The secondary aim is to assess their association with other clinical outcomes. OS was defined as the time from transplantation to all-caused death. Relapses are defined as the presence of leukemic cells constituting more than 5% of the bone marrow or the existence of extramedullary leukemia. Relapse-free survival (RFS) indicates the span of post-transplant survival free from disease relapse. NRM refers to death occurring without prior relapse of the disease. GVHD was diagnosed and classified according to established guidelines (14). CMV and EBV seropositivity was confirmed when DNA copy numbers in peripheral blood reached ≥500 copies/mL on two successive tests.

An absolute neutrophil count of ≥ 0.5 × 10^9^/L maintained for 3 consecutive days was considered neutrophil engraftment. PLT engraftment occurred when PLT counts exceeded 20 × 10^9^/L for 7 consecutive days without requiring transfusions.

### Statistical analysis

A t-test or Mann–Whitney U test was selected to evaluate continuous variables and the χ² or Fisher’s exact test to categorical variables. The visualization of OS and RFS was done using Kaplan–Meier curves, and the log-rank test was applied to evaluate group differences. A Fine and Gray model was applied to assess outcomes affected by competing risks: NRM (competing events: relapse and relapse-related death), relapse (competing event: death), and GVHD (competing event: death). To identify the optimal cutoff value for NRM, the maximally selected log-rank statistics were used. Univariate analysis was carried out by time-dependent Cox regression or a Fine and Gray model. Variables showing a p-value of ≤ 0.1 in the univariate analysis were incorporated into the multivariate analysis. The proportional hazards assumption was assessed using time-by-covariate interaction terms within the Fine–Gray model, and Schoenfeld residuals were examined for cause-specific Cox models. For the primary variable of interest, when a violation of the proportional hazards assumption was detected, a time-dependent effect was incorporated by including an interaction term between the covariate and log(time) in the model. To explore associations between continuous variables, Pearson’s correlation was applied. *P* < 0.05 was considered statistically significant. All analyses and visual representations were generated using R (version 4.3.3) and GraphPad Prism (version 8).

## Results

### Patients’ characteristics

A comparison of baseline patient characteristics between the training and validation cohorts is provided in **Table 1**. MRD positivity, received tacrolimus, as well as CMV and EBV seropositivity, were more common in the training cohort. Apart from these, the training and validation cohorts showed similar baseline profiles (all *P* > 0.05).

**Table 1:** Baseline characteristics of all patients included in this study.

Variable	Training cohort (N = 195)	Validation cohort (N = 109)	P-value
Age (month), median (range)	108.0 (9.0-200.0)	114.0 (9.0-213.0)	0.449
Gender			
Male	122 (62.8%)	69 (63.3%)	0.898
Female	73 (37.4%)	40 (36.7%)	
Disease			0.771
AML	105 (53.8%)	57 (52.3%)	
M1+M2	41	19	
M4+M5	38	22	
M6+M7	5	7	
Not classifiable	20	9	
ALL	82 (42.1%)	49 (45.0%)	
B-ALL	66	39	
T-ALL	16	10	
MDS	8 (4.1%)	3 (2.8%)	
Disease status			0.065
CR	174 (89.2%)	104 (95.4%)	
No-CR	21 (10.8%)	5 (4.6%)	
MRD			0.022*
Positive	35 (17.9%)	9 (8.3%)	
Negative	148 (75.9%)	97 (89%)	
NA	12 (6.2%)	3 (2.8%)	
TBI			0.471
Yes	23 (11.8%)	16 (4.7%)	
No	172 (88.2%)	93 (85.3%)	
ABO match			0.862
Match	104 (53.3%)	57 (52.3%)	
Mismatch	91 (46.7%)	52 (47.7%)	
Doner-patient sex matched			0.148
Female-male	27 (13.8%)	9 (8.3%)	
Others	168 (86.2%)	100 (91.7%)	
HLA compatibility			0.495
5/10	124 (63.6%)	65 (59.6%)	
Others	71 (36.4%)	44 (40.4%)	
Graft source			0.865
PB	59 (30.3%)	34 (31.2%)	
PB+BM	136 (69.3%)	75 (68.8%)	
GVHD prevention			0.033*
CSA + MMF + MTX	180 (92.3%)	107 (98.2%)	
FK506 + MMF + MTX	15 (7.7%)	2 (1.8%)	
AGVHD			0.318
Grade 0-I	127 (65.1%)	79 (72.5%)	
Grade II	35 (17.9%)	18 (16.5%)	
Grade III-IV	33 (16.9%)	12 (11.0%)	
CGVHD			0.255
Positive	66 (33.8%)	30 (27.5%)	
Negative	129 (66.2%)	79 (72.5%)	
CMV seropositive			< 0.001*
Positive	132 (67.7%)	41 (37.6%)	
Negative	63 (32.3%)	68 (62.4%)	
EBV seropositive			< 0.001*
Positive	137 (70.3%)	31 (28.4%)	
Negative	58 (29.7%)	78 (71.6%)	
BSI			0.599
Positive	38 (19.5%)	24 (22.0%)	
Negative	157 (80.4%)	85 (78.0%)	
MNC, ×10^8^/kg	7.1 (0.6-21.7)	6.6 (3.8-17.7)	0.133
CD34, ×10^6^/kg	6.9 (0.9-18.3)	7.3 (3.2-15.0)	0.148
Neutrophil engraftment time (d), median (range)	12.0 (9.0-21.0)	12.0 (9.0-21.0)	0.153
Platelet engraftment time (d), median (range)	11.0 (5.0-40.0)	11.0 (7.0-61.0)	0.769

AML, acute myeloid leukemia; ALL, acute lymphoblastic leukemia; MDS, myelodysplastic syndrome; CR, complete remission; MRD, minimal residual disease; TBI, total body irradiation; HLA, human leukocyte antigen; PB, Peripheral blood; BM, bone marrow; GVHD, graft versus host disease; CSA, cyclosporine A; FK506, tacrolimus; MMF, mycophenolate mofetil; CMV, Cytomegalovirus; EBV, Epstein-Barr virus; BSI, bloodstream infections; MNC, mononuclear cells. *represent statistical significance p < 0.05.

### The relationship between EASIX/s-EASIX/m-EASIX score and clinical outcomes in the training cohort

**Figure 1:**
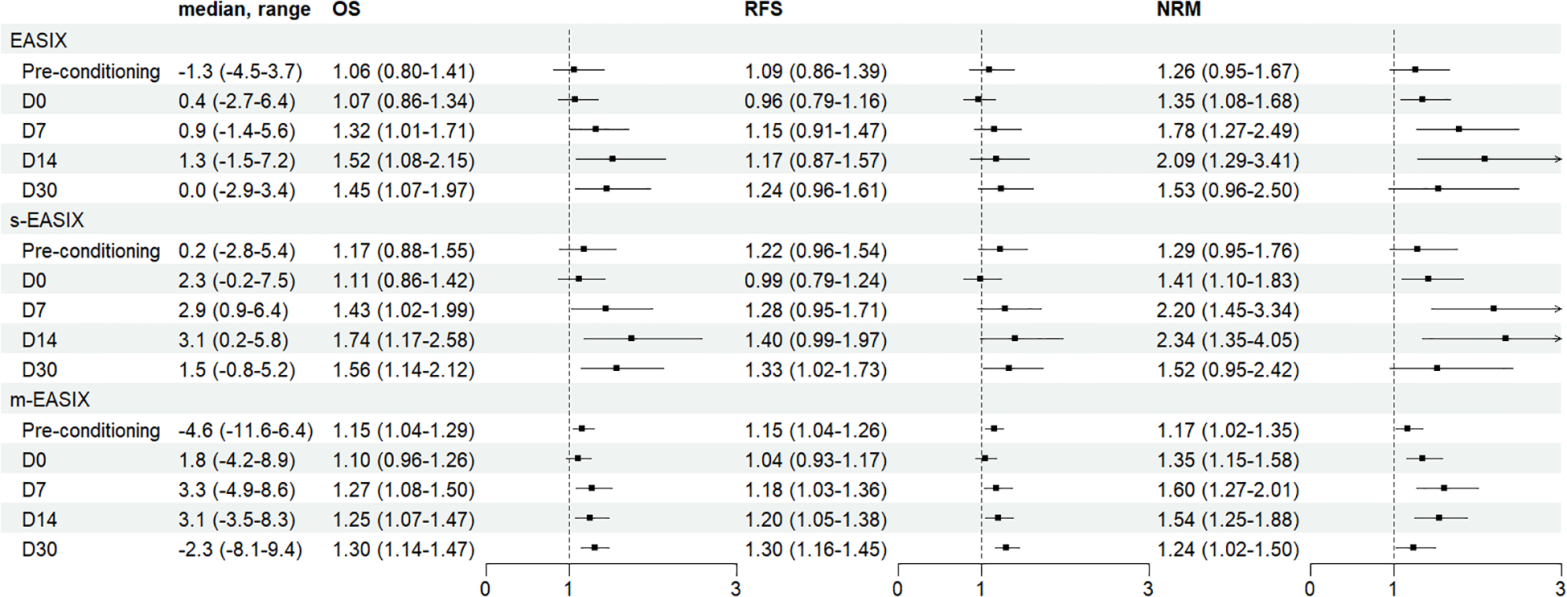
Association of EASIX, s-EASIX and m-EASIX scores and main outcomes in the training cohort (N = 195). This forest plot illustrates the unadjusted hazard ratios (HRs) and their 95% confidence intervals (CIs) for EASIX, s-EASIX, and m-EASIX scores at various time points (Pre-conditioning, D0, D7, D14, D30) in relation to OS, RFS, and NRM. The square markers represent the HR, and the horizontal lines indicate the 95% CI. The figure demonstrates that D7 and D14 m-EASIX scores, among others, show significant associations with OS, RFS, and particularly NRM.

During a median of 35.5 (3.9-59.9) months follow-up, there were 36 deaths. The 3-year OS rate was 82.0% (95% CI: 76.5%–87.8%). All 3 formulas were associated with OS at three time points: D7, D14, and D30 (eg, at D7, EASIX: HR, 1.32; 95% CI, 1.01-1.71; *P* = 0.042; s-EASIX: HR, 1.43; 95% CI, 1.02-1.99; *P* = 0.036; m-EASIX: HR, 1.27; 95% CI, 1.08-1.50; *P* = 0.005). A total of 32 patients experienced relapse, with a 3-year cumulative relapse rate of 14.6%. The probability of 3-year RFS was 75.1% (95% CI: 68.9%-81.7%). M-EASIX was significantly linked to inferior RFS at most timepoints (eg, at D7, HR, 1.18; 95% CI, 1.03-1.36; *P* = 0.017). The cumulative incidence of NRM was 10.4% at 3 years. Elevated values of the 3 formulas at time points other than pre-conditioning and D30 were linked to increased NRM rates (eg, at D7, EASIX: HR, 1.78; 95% CI, 1.27-2.49; *P* < 0.001; s-EASIX: HR, 2.20; 95% CI, 1.45-3.34; *P* < 0.001; m-EASIX: HR, 1.60; 95% CI, 1.27-2.01; *P* < 0.001) (**Figure 1**).

### Contribution of LDH, creatinine, PLT and CRP in the training cohort

Subsequently, the specific contribution of each variable within the formulas was assessed separately. The results can be found in the **Supplementary File (Table S1)**. Among all time points, LDH was associated with NRM on D0 (HR, 1.50; 95% CI, 1.08-2.08; *P* = 0.016) and D7 (HR, 1.91; 95% CI, 1.14-3.20; *P* = 0.014), and with OS on D14 (HR, 1.65; 95% CI, 1.02-2.68; *P* = 0.042). For PLT variable, lower level at D30 were associated with poorer clinical outcomes (eg. OS: HR, 0.67; 95% CI, 0.48-0.93; *P* = 0.017; RFS: HR, 0.64; 95% CI, 0.43-0.97; *P* = 0.034; NRM: HR, 0.64; 95% CI, 0.43-0.97, *P* = 0.034). At D7, CRP levels showed a consistent correlation with OS, RFS and NRM (OS: HR, 1.28; 95% CI, 1.04-1.56; *P* = 0.018; RFS: HR, 1.19 95% CI, 1.01-1.41; *P* = 0.039; NRM: HR, 1.42; 95% CI, 1.11-1.82; *P* = 0.005). In contrast, creatinine was only related to RFS at the preconditioning (HR, 0.46 95% CI, 0.24-0.91; *P* = 0.025) and showed no association with clinical outcomes at other time points.

Considering that LDH, CRP, and PLT were associated with OS, RFS, and NRM at D7, we chose D7-m-EASIX for further analysis.

### D7-m-EASIX and clinical outcomes following HSCT in the training cohort

Based on the optimal cutoff determined by the maximally selected log-rank statistics (cutoff: 4.1), the cohort was categorized into high and low D7-m-EASIX groups. After excluding one patient who died before day 7, 194 patients were analyzed, with 139 in the low D7-m-EASIX group and 55 in the high D7-m-EASIX group. Compared to the low D7-m-EASIX group, the high D7-m-EASIX group had an older age (*P* = 0.023) and lower CD34^+^ cell dose (*P* = 0.001, **Supplementary Table S2**).

Since only gene mutation and fusion data were available for pediatric AML patients, this part of the analysis focused solely on the AML cohort. We generated waterfall plots of gene mutations and fusions and compared differences between the high and low EASIX groups (**Supplementary Figure S1**). The results showed that, in the training cohort, the most common mutations in both high and low EASIX groups were *FLT3*, *NRAS*, *WT1*, and *CEBPA*, while the most frequent fusion genes were *RUNX1–RUNX1T1*, *MLL–AF9*, and *CBFB–MYH11* (all *P*>0.05).

**Figure 2:**
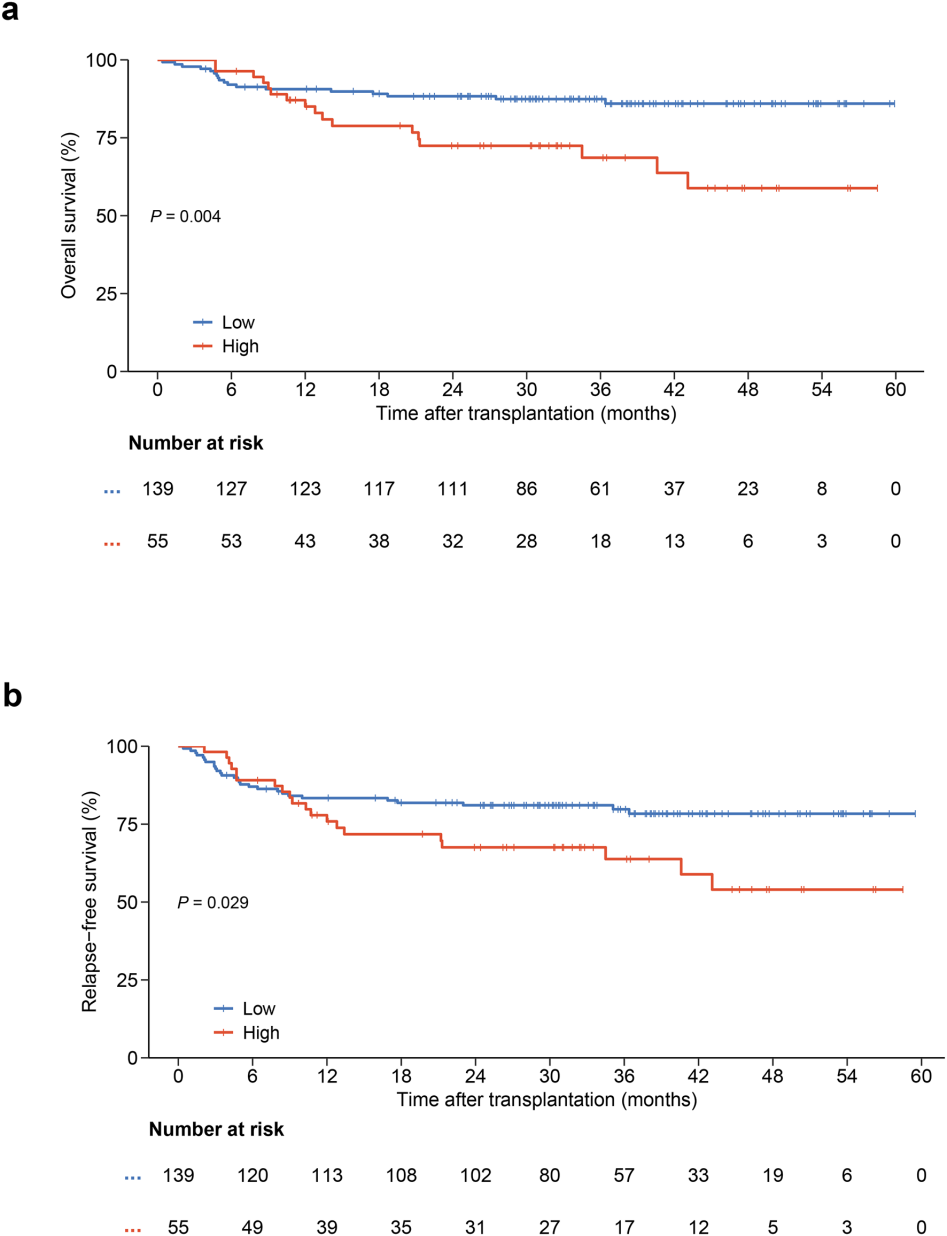
Overall survival and Relapse-free survival according to D7-m-EASIX score in the training cohort (High group: n = 55; Low group: n = 139). **(a)** overall survival; **(b)** relapse-free survival. Survival curves were estimated by the Kaplan–Meier method and compared using the log-rank test. Patients with higher D7-m-EASIX scores had significantly worse OS and RFS.

**Figure 3:**
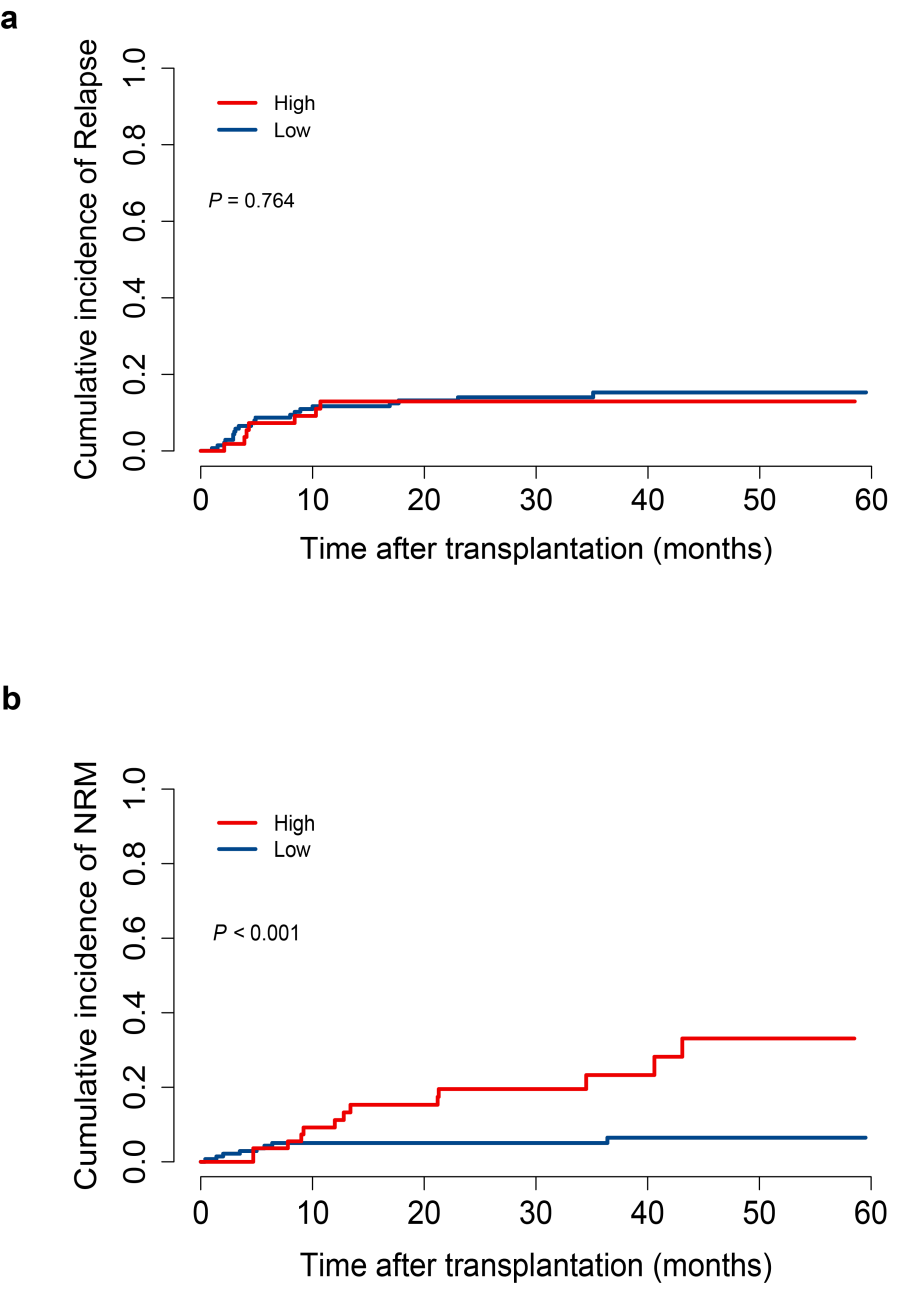
The cumulative incidences of relapse and non-relapse morality according to D7-m-EASIX score in the training cohort (High group: n = 55; Low group: n = 139). **(a)** relapse; **(b)** non-relapse morality. Cumulative incidence curves were estimated using the Fine–Gray method. Higher D7-m-EASIX was associated with increased NRM.

Patients with a higher D7-m-EASIX score had a worse 3-year OS (68.6% ± 7.0% VS 87.4% ± 2.9%, *P* = 0.004, **Figure 2A**) and 3-year RFS (63.8% ± 7.2% VS 79.8% ± 3.5%, *P* = 0.029, **Figure 2B**). To determine whether the D7-m-EASIX score is an independent prognostic factor, the time-dependent Cox regression analysis was utilized. In the univariate analysis, higher D7-m-EASIX score (*P* = 0.005), lower dose of CD34+ cell (*P* = 0.011) and bloodstream infections (BSI, *P* = 0.002) were associated with poorer OS (**Table 2**). Additionally, non-complete remission (non-CR, *P* = 0.022), BSI (*P* = 0.011), minimal residual disease positive (MRD, *P* = 0.022), higher D7-m-EASIX score (*P* = 0.032) and lower dose of CD34+ cell (*P* = 0.029) predict worse RFS (**Table 2**). Variables with *P* ≤ 0.1 were subsequently entered into the multivariate analysis, which identified the D7-m-EASIX score as the sole independent predictor of OS and RFS (HR: 2.35; 95%CI, 1.19-4.62. *P* = 0.013 and HR: 1.85; 95%CI, 1.01-3.39. *P* = 0.047, respectively **Table 3**). Schoenfeld residuals indicated that D7-m-EASIX violated the proportional hazards assumption (*P* < 0.05). Therefore, D7-m-EASIX was modeled with a log(time) interaction, showing an increasing hazard over time for OS and RFS (**Supplementary Figure S2**).

**Table 2:** Univariate analysis of risk factors for main outcomes in the training cohort.

Variable	OS			RFS			NRM		
	HR	95% CI	*P* value	HR	95% CI	*P* value	HR	95% CI	*P* value
Sex (male VS female)	0.96	0.49-1.87	0.902	0.84	0.48-1.49	0.549	2.18	0.81-5.90	0.125
Age	1.00	0.99-1.01	0.978	1.00	0.99-1.01	0.671	1.01	1.00-1.02	0.254
Disease (AML & MDS VS ALL)	0.73	0.38-1.40	0.340	0.88	0.50-1.55	0.658	0.51	0.22-1.18	0.116
Disease status (non-CR VS CR)	1.01	0.36-2.85	0.992	2.24	1.12-4.51	0.022*	0.35	0.04-2.74	0.316
MRD (positive VS negative)	1.55	0.72-3.30	0.259	2.18	1.18-4.01	**0.013**	0.99	0.33-2.94	0.982
TBI (yes VS no)	1.07	0.38-3.04	0.895	0.94	0.37-2.38	0.900	0.84	0.19-3.65	0.817
Blood-type match (mismatched *vs* matched)	0.82	0.48-1.58	0.548	0.78	0.44-1.39	0.404	0.52	0.21-1.29	0.159
Donor-recipient sex match (female to male *vs* others)	1.50	0.66-3.42	0.338	0.99	0.44-2.20	0.976	2.38	0.99-5.76	0.054*
Graft source (PB+BM *vs* PB)	0.55	0.28-1.09	0.085*	0.69	0.38-1.25	0.221	0.52	0.23-1.19	0.119
HLA compatibility (5/10 *vs* others)	0.93	0.47-1.84	0.839	0.88	0.49-1.58	0.666	0.75	0.31-1.83	0.531
Graft Dose									
MNC	0.94	0.82-1.07	0.317	0.93	0.83-1.04	0.187	0.87	0.73-1.05	0.153
CD34	0.83	0.72-0.96	0.011*	0.88	0.78-0.99	0.029*	0.70	0.55-0.89	0.004*
CMV (positive VS negative)	1.18	0.57-2.45	0.654	1.17	0.63-2.18	0.618	1.18	0.46-3.03	0.725
EBV (positive VS negative)	0.93	0.45-1.94	0.844	1.05	0.56-1.99	0.873	0.45	0.20-1.11	0.087*
BSI (positive VS negative)	2.86	1.46-5.60	0.002*	2.17	1.19-3.94	0.011*	1.95	0.80-4.72	0.139
D7-m-EASIX (high VS low)	2.57	1.32-4.98	0.005*	1.88	1.06-3.33	0.032*	4.38	1.81-10.61	0.001*

OS, overall survival; RFS, relapse-free survival; NRM, non-relapse mortality. AML, acute myeloid leukemia; ALL, acute lymphoblastic leukemia; MDS, myelodysplastic syndrome; CR, complete remission; MRD, minimal residual disease; TBI, total body irradiation; PB, Peripheral blood; BM, bone marrow; HLA, human leukocyte antigen; MNC, mononuclear cells; CMV, Cytomegalovirus; EBV, Epstein-Barr virus; BSI, bloodstream infections.

*represent statistical significance p ≤ 0.10.

**Table 3:** Multivariate analysis of risk factors for main outcomes in the training cohort.

Variable	OS			RFS			NRM		
	HR	95% CI	*P* value	HR	95% CI	*P* value	HR	95% CI	*P* value
Disease status (non-CR VS CR)				1.50	0.50-4.53	0.467			
MRD (positive VS negative)				1.68	0.64-4.43	0.291			
Donor-recipient sex match (female to male *vs* others)							1.49	0.50-4.41	0.480
Graft source (PB+BM *vs* PB)	0.71	0.35-1.45	0.351						
CD34	0.92	0.80-1.05	0.210	0.95	0.85-1.07	0.432	0.81	0.63-1.03	0.089
BSI (positive VS negative)	2.76	1.39-5.47	0.004*	2.00	1.07-3.74	0.030*			
EBV (positive VS negative)							0.59	0.23-1.53	0.280
D7-m-EASIX (high VS low)	2.35	1.19-4.62	0.013*	1.85	1.01-3.39	0.047*	3.32	1.35-8.18	0.009*

OS, overall survival; RFS, relapse-free survival; NRM, non-relapse mortality. CR, complete remission; MRD, minimal residual disease; PB, Peripheral blood; BM, bone marrow; BSI, bloodstream infections. EBV; Epstein-Barr virus.

*represent statistical significance p < 0.05.

Relapse occurred at similar rates in both the low and high D7-m-EASIX groups (15.3% VS 12.9%, *P* = 0.764, **Figure 3A**). However, patients with elevated D7-m-EASIX scores exhibited a higher rate of NRM (23.3% VS 5.1%, *P* < 0.001, **Figure 3B**). In the multivariate Fine-Gray test, A higher D7-m-EASIX score increased the risk of NRM (HR: 3.32; 95%CI, 1.35-8.18. *P* = 0.010, respectively **Table 3**). Additionally, time-by-covariate interaction analyses within the Fine–Gray model indicated that the primary variable D7-m-EASIX satisfied the proportional hazards assumption (*P*>0.05).

The leading causes of death in the low D7-m-EASIX group were relapse, infection and GVHD, whereas in the high D7-m-EASIX group, they were infection, GVHD and relapse (**Supplementary Table S3**).

Considering the potential impact of PLT transfusion on the D7-m-EASIX score, we examined the cumulative dose of PLT transfusion administered from the start of conditioning to before D7. The high D7-m-EASIX group tended to receive PLT transfusions on D6 (*P* = 0.187). Additionally, the high D7-m-EASIX group required higher mean platelet transfusions (3.5unite *vs*. 3.0 unite, *P* = 0.028). After categorizing PLT transfusions into tertiles, the D7-m-EASIX score was highest in the highest tertile (3.8 [–4.9 to 8.6] VS 3.2 [–4.4 to 7.2] VS. 1.8 [–1.2 to 6.0], *P* = 0.005). Additionally, we included both day 6 platelet transfusion (D6, yes/no) in multivariate models. We found that D7-m-EASIX remained an independent predictor for OS and NRM (*P* < 0.05), while for RFS, the effect became borderline (*P* = 0.085).

**Figure 4:**
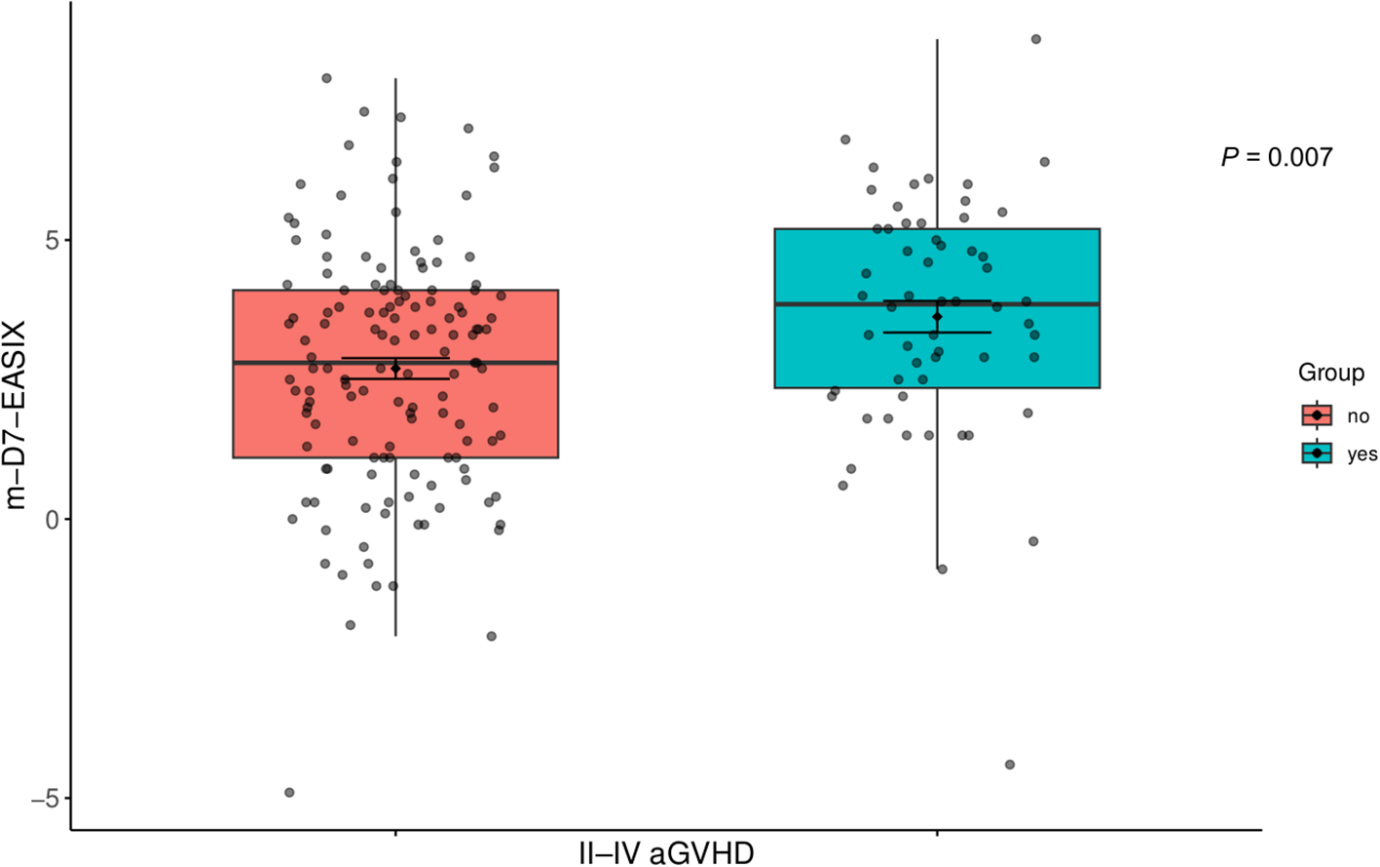
Distribution of D7-m-EASIX scores across II-IV aGVHD in the training cohort (aGVHD: n = 58; normal: n = 136). Each box represents the interquartile range, with the horizontal line inside indicating the median D7-m-EASIX score. The black dots and vertical lines within the boxes represent the mean and standard deviation, respectively. Statistical comparison between the two groups was performed using the the Mann–Whitney U test. Boxplots display higher m-EASIX levels in patients with II-IV aGVHD.

**Figure 5:**
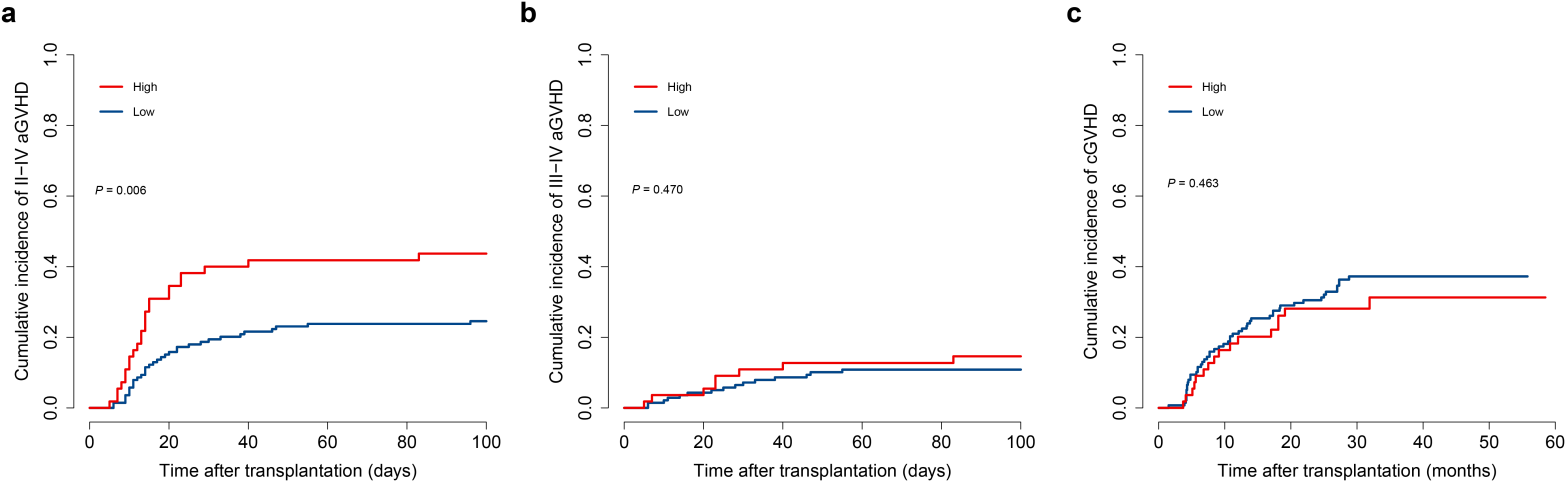
The impact of D7-m-EASIX score on GVHD in the training cohort. **(a)** cumulative incidence of II-IV aGVHD; **(b)** cumulative incidence of III-IV aGVHD; **(c)** cumulative incidence of cGVHD. Competing-risk analyses were performed using Fine–Gray models, with death as the competing event. P values were calculated using the Gray’s test. Patients with a high D7-m-EASIX score showed a significantly higher incidence of II–IV aGVHD (*P* = 0.005), whereas no significant difference was observed for III–IV aGVHD or chronic GVHD.

II–IV aGVHD occurred in 58 patients, emerging at a median of 14 days (range: 5–96) post-HSCT. Patients with grade II–IV aGVHD demonstrated markedly elevated D7-m-EASIX scores (3.8 [–4.4 to 8.6] VS. 2.8 [–4.9 to 7.9], *P* = 0.007, **Figure 4**). The group with lower D7-m-EASIX scores showed a reduced cumulative incidence of II–IV aGVHD (24.7% VS 43.7%, *P* = 0.006, **Figure 5A**). Multivariate analysis revealed the D7-m-EASIX score was associated with increased risk of II–IV aGVHD (HR: 2.16; 95%CI, 1.30-3.57. *P* = 0.003, **Table 4**). Additionally, the primary variable D7-m-EASIX satisfied the proportional hazards assumption (*P*>0.05). When only focus on III-IV aGVHD, no statistically significant variation was found between the groups (*P* = 0.470, **Figure 5B**). Likewise, both groups exhibited comparable rates of chronic GVHD (*P* = 0.463, **Figure 5C**).

**Table 4:** Univariate analysis and multivariate analysis of II-IV aGVHD in the training cohort.

Variable	Univariate analysis			Multivariate analysis		
	HR	95% CI	*P* value	HR	95% CI	*P* value
Sex (male VS female)	0.98	0.58-1.66	0.947			
Age	1.00	0.99-1.00	0.139			
Disease (AML & MDS VS ALL)	1.07	0.63-1.81	0.795			
Disease status (non-CR VS CR)	0.54	0.21-1.39	0.201			
MRD (positive VS negative)	0.59	0.27-1.25	0.169			
TBI (positive VS negative)	0.86	0.37-2.01	0.721			
Blood-type match (mismatched *vs* matched)	0.82	0.49-1.36	0.438			
Donor-recipient sex match (female to male *vs* others)	1.63	0.85-3.16	0.145			
Graft source (PB+BM *vs* PB)	0.84	0.49-1.46	0.543			
HLA compatibility (5/10 *vs* others)	0.87	0.50-1.50	0.605			
Graft Dose						
MNC	1.03	0.96-1.10	0.456			
CD34	0.98	0.90-1.07	0.723			
CMV (positive VS negative)	0.79	0.48-1.32	0.370			
EBV (positive VS negative)	0.96	0.57-1.61	0.871			
BSI (positive VS negative)	1.91	1.09-3.32	0.021*	1.98	1.16-3.39	0.013*
D7-m-EASIX (high VS low)	2.09	1.24-3.52	0.005*	2.16	1.30-3.57	0.003*

GVHD, graft-versus-host disease. AML, acute myeloid leukemia; ALL, acute lymphoblastic leukemia; MDS, myelodysplastic syndrome; CR, complete remission; MRD, minimal residual disease; TBI, total body irradiation; PB, Peripheral blood; BM, bone marrow; HLA, human leukocyte antigen; MNC, mononuclear cells; CMV, Cytomegalovirus; EBV, Epstein-Barr virus; BSI, bloodstream infections.

* represent statistical significance p ≤ 0.10 in univariate analysis and p < 0.05 in multivariate analysis.

We further explored the role of m-EASIX at the onset of II–IV aGVHD. The median II-IV aGVHD-mEASIX was 2.6 (-5.1-8.3). As a continuous variable, II-IV aGVHD-mEASIX had no impact on OS, RFS, or NRM (all *P* > 0.05). Similarly, III-IV aGVHD-mEASIX had no impact on prognosis (all *P* > 0.05).

### Sensitivity analysis

To assess potential confounding from acute inflammation, we performed a sensitivity analysis excluding patients with CRP ≥ 50 mg/L or documented infection, leaving 172 patients for analysis. The results showed that patients with high D7-m-EASIX had lower 3-year OS (66.4% ± 8.6% *vs*. 86.9% ± 3.0%, P = 0.005) and RFS (59.6% ± 8.7% *vs*. 79.0% ± 3.7%, P = 0.017), and higher NRM (24.4% *vs*. 5.2%, P < 0.001), supporting the robustness of our findings.

### The relationship between D7-m-EASIX score and age in the training cohort

**Figure 6:**
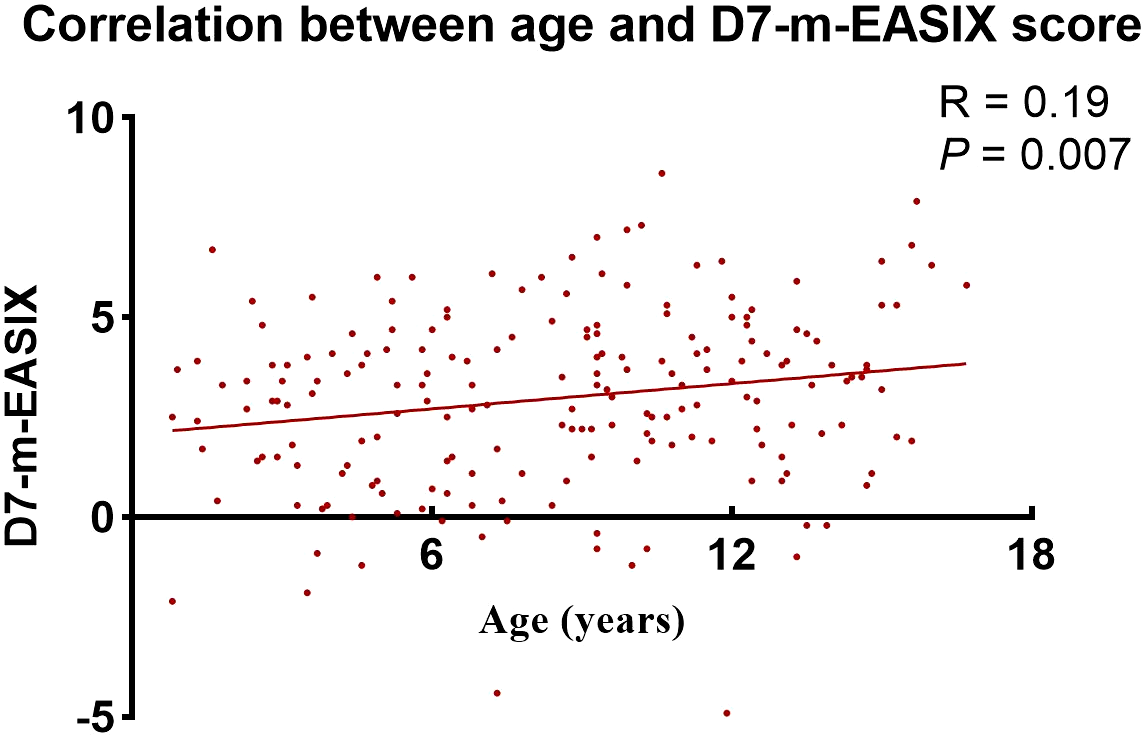
The correlation between age and D7-m-EASIX score in the training cohort. A scatter plot displays the relationship between patient age and D7-m-EASIX score. A linear regression line (in red) is superimposed to illustrate the general trend. The correlation was assessed using Spearman’s rank correlation, revealing a statistically significant but weak positive association.

To evaluate the association between the D7-m-EASIX score and age, the Spearman rank correlation coefficient was applied. The results indicated a slightly positive relationship (R = 0.19, *P* = 0.007, **Figure 6**). However, when age was included in the multivariate analysis, D7-m-EASIX (as a categorical variable) remained an independent predictor for OS, RFS, NRM, and was associated with II–IV aGVHD (all *P* < 0.05).

### D7-m-EASIX and clinical outcomes following HSCT in the validation cohort

Similarly, using a cutoff of 4.1, classification into high and low D7-m-EASIX groups was applied to patients in the validation cohort (**Supplementary Table S2**). We further compared gene mutations and fusions between the high and low EASIX groups and found that *AML–ETO* was more frequent in the high group (*P* < 0.05), with no other significant differences (**Supplementary Figure S1**).

A comparable pattern was observed, as in the training cohort. The high D7-m-EASIX group showed significantly lower 2-year OS (63.4% ± 11.7% VS 93.6% ± 3.2%, *P* < 0.001) and RFS (64.1% ± 11.5% VS 82.8% ± 5.6%, *P* = 0.019), and higher NRM (26.6% VS 3.1%, *P* = 0.002) and grade II–IV aGVHD rates (50.0% VS 20.0%, *P* = 0.005). Additionally, we found that D7-m-EASIX > 4.1 increased in the incidence of III-IV aGVHD (12.5% VS 3.5%, *P* = 0.025). In multivariate analysis, D7-m-EASIX >4.1 remained an independent factor for adverse clinical outcomes and satisfied the proportional hazards assumption (**Supplementary Tables S4–S6**; **Supplementary Figures S3–S5**). In addition, when day 6 platelet transfusion (D6, yes/no) was included in the model, D7-m-EASIX remained an independent predictor (all *P* < 0.05).

## Discussion

A major concern after HSCT is NRM. Previous studies have shown that EASIX is linked to higher NRM and an increased incidence of endothelium-related complications. However, this association has not yet been well validated in pediatric haploidentical transplant cohorts.

The EASIX formula is composed of LDH, creatinine, and PLT count, which are the most common laboratory indicators associated with endothelial dysfunction (3, 15). During endothelial injury, elevated LDH levels are attributed to the release from damaged endothelial cells and circulating cells such as leukocytes and PLTs (16). Endothelial dysfunction contributes significantly to the development of conditions like acute and chronic kidney disease (17, 18). When renal endothelial injury occurs, the regulation of vascular tone becomes imbalanced, especially due to reduced availability of nitric oxide, resulting in decreased renal perfusion and increased creatinine levels (19). Low PLTs are also a consequence of endothelial injury. During severe vascular damage, endothelial cell death or denudation leads to exposure of the underlying extracellular matrix, which activates and binds circulating platelets. This interaction promotes microthrombus formation, resulting in significant platelet consumption (20). CRP is a marker of acute inflammation, synthesized by the liver in response to pro-inflammatory cytokines. Elevated CRP levels have been linked to impaired endothelial vascular reactivity (21).

The predictive value of EASIX at different time points has been well demonstrated in adult cohorts. Penack et al. found that patients with a pre-conditioning EASIX score ≥3 had more than twofold increased risk of NRM in a multicenter prospective study (5). Additionally, the EASIX score at 1 year post-transplant can also help identify patients at high risk of NRM (22). In the pediatric cohort, Luft et al. demonstrated that pre-conditioning EASIX score was linked to OS and NRM only in univariate analysis (4). However, in a subsequent study, Muratore et al. identified D7- EASIX as an independent predictor of SOS and NRM (9). Our study also revealed that a D7-m-EASIX score >4.1 increased the risk of NRM. The discrepancy could be clarified by the following points: (a) the cohort in Luft et al’s study primarily received reduced-intensity conditioning, whereas both Muratore et al. and our study focused on patients undergoing MAC. (b) Luft et al. evaluated EASIX at the pre-conditioning time point. Given that endothelial function can be affected by multiple elements such as the conditioning regimen, the transplantation process itself, and infections (23), post-transplant EASIX might serve as a more accurate predictor of NRM than pre-conditioning measurements (24). In the adult cohort reported by Luft et al., the individual components of EASIX—LDH, creatinine, and platelet count—were each evaluated for their association with NRM, and all three components showed a significant correlation with adverse outcomes (4). In contrast, in our pediatric cohort, creatinine levels at any time point were not associated with NRM. This discrepancy may be attributed to age-related physiological differences, including lower baseline renal function decline, higher nitric oxide bioavailability, and differences in the systemic renin–angiotensin system activity in children compared with adults (25).

We found that a D7-m-EASIX score >4.1 was related to higher incidence of II-IV aGVHD. Suppression of tumorigenicity 2 (ST2) is currently one of the most promising biomarkers for aGVHD. ST2 levels on day 28 post-transplantation can predict the occurrence of aGVHD and transplant-related mortality (26). Moreover, ST2 levels at the onset of aGVHD show a strong link to steroid-refractory aGVHD and NRM (27). However, Luft et al. found no correlation between ST2 levels and pre-conditioning EASIX (4). In contrast, for the biomarker tumor necrosis factor 1, Pedraza observed a parallel trend with EASIX scores and identified both as independent predictors of aGVHD (28). Unfortunately, due to limitations in this retrospective study, we were unable to validate the association between aGVHD biomarkers and m-EASIX score.

In our study, GVHD-m-EASIX failed to predict clinical outcomes in patients with aGVHD. All included patients in our study received MAC conditioning, which differs from previous studies. These studies typically suggest that GVHD-EASIX serves as a meaningful prognostic marker for aGVHD in patients who primarily receive reduced-intensity conditioning (29, 30). One possible explanation for this discrepancy is that patients undergoing MAC conditioning tend to have lower platelet counts at the time of aGVHD onset, which may influence the predictive value of GVHD-m-EASIX in this population (29).

This research is limited by several factors that warrant further exploration in future studies. First, a retrospective and observational study conducted at a single center, the applicability of the results might be restricted. Second, given the limited cases of SOS in our study, we were unable to further evaluate the potential predictive value of m-EASIX for SOS. Third, although a positive correlation was observed between age and the D7-m-EASIX score, the correlation coefficient was relatively low (R = 0.19). Fourth, as gene information was limited to AML patients, the potential relationship between ALL molecular features and the D7-m-EASIX score could not be explored. Finally, the retrospective design restricted our ability to explore the relationship between m-EASIX and aGVHD-associated biomarkers.

## Conclusion

In conclusion, this study suggests that D7-m-EASIX could be a valuable marker for assessing prognosis in pediatric patients receiving MAC-based HID transplantation. In future clinical practice, D7-m-EASIX holds potential as a useful tool for identifying children at high risk of NRM, thereby supporting early intervention and personalized management strategies.

## Data Availability

The raw data supporting the conclusions of this article will be made available by the authors, without undue reservation.

## References

[1] Carreras E Diaz-Ricart M . The role of the endothelium in the short-term complications of hematopoietic SCT. Bone Marrow Transplant. (2011) 46:1495–502. doi: 10.1038/bmt.2011.65, PMID: 21460864

[2] Cordes S Mokhtari Z Bartosova M Mertlitz S Riesner K Shi Y . Endothelial damage and dysfunction in acute graft-versus-host disease. Haematologica. (2021) 106:2147–60. doi: 10.3324/haematol.2020.253716, PMID: 32675225 PMC8327719

[3] Ruutu T Barosi G Benjamin RJ Clark RE George JN Gratwohl A . Diagnostic criteria for hematopoietic stem cell transplant-associated microangiopathy: results of a consensus process by an International Working Group. Haematologica. (2007) 92:95–100. doi: 10.3324/haematol.10699, PMID: 17229640

[4] Luft T Benner A Terzer T Jodele S Dandoy CE Storb R . EASIX and mortality after allogeneic stem cell transplantation. Bone Marrow Transplant. (2020) 55:553–61. doi: 10.1038/s41409-019-0703-1, PMID: 31558788 PMC8082940

[5] Penack O Luft T Peczynski C Benner A Sica S Arat M . Endothelial Activation and Stress Index (EASIX) to predict mortality after allogeneic stem cell transplantation: a prospective study. J Immunother Cancer. (2024) 12. doi: 10.1136/jitc-2023-007635, PMID: 38199608 PMC10806535

[6] Shouval R Fein JA Shouval A Danylesko I Shem-Tov N Zlotnik M . External validation and comparison of multiple prognostic scores in allogeneic hematopoietic stem cell transplantation. Blood Adv. (2019) 3:1881–90. doi: 10.1182/bloodadvances.2019032268, PMID: 31221661 PMC6595255

[7] Jiang S Penack O Terzer T Schult D Majer-Lauterbach J Radujkovic A . Predicting sinusoidal obstruction syndrome after allogeneic stem cell transplantation with the EASIX biomarker panel. Haematologica. (2021) 106:446–53. doi: 10.3324/haematol.2019.238790, PMID: 31974195 PMC7849560

[8] Sanchez-Escamilla M Flynn J Devlin S Maloy M Fatmi SA Tomas AA . EASIX score predicts inferior survival after allogeneic hematopoietic cell transplantation. Bone Marrow Transplant. (2023) 58:498–505. doi: 10.1038/s41409-023-01922-8, PMID: 36721042 PMC10513445

[9] Muratore E Gambuti G Leardini D Baccelli F Venturelli F Larcinese L . The EASIX score as a predictor of sinusoidal obstruction syndrome and nonrelapse mortality in paediatric patients receiving allogeneic haematopoietic stem cell transplantation. Bone Marrow Transplant. (2025) 60:346–52. doi: 10.1038/s41409-024-02489-8, PMID: 39658654 PMC11893459

[10] Kunacheewa C Ungprasert P Phikulsod P Issaragrisil S Owattanapanich W . Comparative efficacy and clinical outcomes of haploidentical stem cell transplantation to other stem sources for treatment in acute myeloid leukemia and myelodysplastic syndrome patients: A systematic review and meta-analysis. Cell Transplant. (2020) 29:963689720904965. doi: 10.1177/0963689720904965, PMID: 32323567 PMC7444220

[11] Wu H Zhao Y Gao F Shi J Luo Y Yu J . Haploidentical transplants deliver equal outcomes to matched sibling transplants: a propensity score-matched analysis. J Transl Med. (2023) 21:329. doi: 10.1186/s12967-023-04168-6, PMID: 37198603 PMC10193779

[12] Konuma T Monna-Oiwa M Kato S Andoh S Isobe M Nannya Y . Levels of C-reactive protein and body temperature elevation during neutropenia predict engraftment and non-relapse mortality for unrelated single-unit cord blood transplantation in adults. Transplant Cell Ther. (2024) 30:1104.e1–.e14. doi: 10.1016/j.jtct.2024.09.008, PMID: 39270934

[13] Pennisi M Sanchez-Escamilla M Flynn JR Shouval R Alarcon Tomas A Silverberg ML . Modified EASIX predicts severe cytokine release syndrome and neurotoxicity after chimeric antigen receptor T cells. Blood Adv. (2021) 5:3397–406. doi: 10.1182/bloodadvances.2020003885, PMID: 34432870 PMC8525234

[14] Harris AC Young R Devine S Hogan WJ Ayuk F Bunworasate U . International, multicenter standardization of acute graft-versus-host disease clinical data collection: A report from the Mount Sinai Acute GVHD international consortium. Biol Blood Marrow Transplant. (2016) 22:4–10. doi: 10.1016/j.bbmt.2015.09.001, PMID: 26386318 PMC4706482

[15] Schoettler ML Carreras E Cho B Dandoy CE Ho VT Jodele S . Harmonizing definitions for diagnostic criteria and prognostic assessment of transplantation-associated thrombotic microangiopathy: A report on behalf of the European society for blood and marrow transplantation, American society for transplantation and cellular therapy, Asia-pacific blood and marrow transplantation group, and center for International blood and marrow transplant research. Transplant Cell Ther. (2023) 29:151–63. doi: 10.1016/j.jtct.2022.11.015, PMID: 36442770 PMC10119629

[16] Chopra J Joist JH Webster RO . Loss of 51chromium, lactate dehydrogenase, and 111indium as indicators of endothelial cell injury. Lab Invest. (1987) 57:578–84., PMID: 3682767

[17] Basile DP . The endothelial cell in ischemic acute kidney injury: implications for acute and chronic function. Kidney Int. (2007) 72:151–6. doi: 10.1038/sj.ki.5002312, PMID: 17495858

[18] Malyszko J . Mechanism of endothelial dysfunction in chronic kidney disease. Clin Chim Acta. (2010) 411:1412–20. doi: 10.1016/j.cca.2010.06.019, PMID: 20598675

[19] Roumeliotis S Mallamaci F Zoccali C . Endothelial dysfunction in chronic kidney disease, from biology to clinical outcomes: A 2020 update. J Clin Med. (2020) 9. doi: 10.3390/jcm9082359, PMID: 32718053 PMC7465707

[20] Dehghani T Panitch A . Endothelial cells, neutrophils and platelets: getting to the bottom of an inflammatory triangle. Open Biol. (2020) 10:200161. doi: 10.1098/rsob.200161, PMID: 33050789 PMC7653352

[21] Devaraj S Yun JM Adamson G Galvez J Jialal I . C-reactive protein impairs the endothelial glycocalyx resulting in endothelial dysfunction. Cardiovasc Res. (2009) 84:479–84. doi: 10.1093/cvr/cvp249, PMID: 19620133 PMC2777951

[22] Kordelas L Terzer T Gooley T Davis C Sandmaier BM Sorror M . EASIX-1year and late mortality after allogeneic stem cell transplantation. Blood Adv. (2023) 7:5374–81. doi: 10.1182/bloodadvances.2022008617, PMID: 37477588 PMC10509665

[23] Palomo M Diaz-Ricart M Carbo C Rovira M Fernandez-Aviles F Escolar G . The release of soluble factors contributing to endothelial activation and damage after hematopoietic stem cell transplantation is not limited to the allogeneic setting and involves several pathogenic mechanisms. Biol Blood Marrow Transplant. (2009) 15:537–46. doi: 10.1016/j.bbmt.2009.01.013, PMID: 19361745

[24] Nawas MT Sanchez-Escamilla M Devlin SM Maloy MA Ruiz JD Sauter CS . Dynamic EASIX scores closely predict nonrelapse mortality after allogeneic hematopoietic cell transplantation. Blood Adv. (2022) 6:5898–907. doi: 10.1182/bloodadvances.2022007381, PMID: 35977079 PMC9661383

[25] Weinstein JR Anderson S . The aging kidney: physiological changes. Adv Chronic Kidney Dis. (2010) 17:302–7. doi: 10.1053/j.ackd.2010.05.002, PMID: 20610357 PMC2901622

[26] Ponce DM Hilden P Mumaw C Devlin SM Lubin M Giralt S . High day 28 ST2 levels predict for acute graft-versus-host disease and transplant-related mortality after cord blood transplantation. Blood. (2015) 125:199–205. doi: 10.1182/blood-2014-06-584789, PMID: 25377785 PMC4281828

[27] Verbeek AB von Asmuth EGJ van den Akker EB Jansen-Hoogendijk AM Schilham MW Lankester AC . Pre-transplant inflammation and its associations with acute GvHD and mortality in pediatric allogeneic hematopoietic stem cell transplantation patients. Bone Marrow Transplant. (2025) 60:948–55. doi: 10.1038/s41409-025-02583-5, PMID: 40234725

[28] Pedraza A Salas MQ Rodríguez-Lobato LG Escribano-Serrat S Suárez-Lledo M Martínez-Cebrian N . Easix score correlates with endothelial dysfunction biomarkers and predicts risk of acute graft-versus-host disease after allogeneic transplantation. Transplant Cell Ther. (2024) 30:187.e1–.e12. doi: 10.1016/j.jtct.2023.11.016, PMID: 38000709

[29] Luft T Benner A Jodele S Dandoy CE Storb R Gooley T . EASIX in patients with acute graft-versus-host disease: a retrospective cohort analysis. Lancet Haematol. (2017) 4:e414–e23. doi: 10.1016/S2352-3026(17)30108-4, PMID: 28733186

[30] Mariotti J Magri F Giordano L De Philippis C Sarina B Mannina D . EASIX predicts non-relapse mortality after haploidentical transplantation with post-transplant cyclophosphamide. Bone Marrow Transplant. (2023) 58:247–56. doi: 10.1038/s41409-022-01874-5, PMID: 36414698

