# Supplementary material for: Modified EASIX score on day 7 predicts survival and non-relapse mortality in pediatric acute leukemia undergoing haploidentical stem cell transplantation
Source: Front Immunol. 2026 Jan 12;16:1646640. doi: 10.3389/fimmu.2025.1646640 (PMC12832470; doi:10.3389/fimmu.2025.1646640)

**Modified EASIX Score on Day 7 Predicts Survival and Non-Relapse Mortality in Pediatric Acute Leukemia Undergoing Haploidentical Stem Cell Transplantation**

Kai Cui^#^, Senlin Zhang^#^, Yueke Du^#^, Yutan Chai, LiGao, Yixin Hu, Bohan Li, Yuanyuan Tian, Yongping Zhang, Shuiyan Wu^*^, Shaoyan Hu^*^, Jie Li^*^

**Supplementary Table 1**. Association of individual variables included in EASIX scores and main outcomes.

| Variable | Median (range) | OS | | |  | RFS | | |  | NRM | | |
| --- | --- | --- | --- | --- | --- | --- | --- | --- | --- | --- | --- | --- |
|  |  | HR | 95% CI | *P* value |  | HR | 95% CI | *P* value |  | HR | 95% CI | *P* value |
| Pre-conditioning |  |  |  |  |  |  |  |  |  |  |  |  |
| LDH (U/L) | 237.8 (130.5, 1019.2) | 1.25 | 0.58-2.69 | 0.569 |  | 0.98 | 0.49-1.95 | 0.947 |  | 1.69 | 0.58-4.94 | 0.334 |
| Creatinine (mg/dL) | 0.3 (0.1, 0.9) | 0.49 | 0.22-1.09 | 0.082 |  | 0.46 | 0.24-0.91 | **0.025*** |  | 0.89 | 0.36-2.23 | 0.803 |
| PLT (10^9^ cells/L) | 210.0 (6.0-1529.0) | 0.87 | 0.64-1.18 | 0.366 |  | 0.80 | 0.63-1.03 | 0.079 |  | 0.81 | 0.57-1.16 | 0.245 |
| CRP (mg/L) | 0.3 (0.0-84.9) | 1.19 | 1.04-1.36 | **0.013*** |  | 1.16 | 1.03-1.31 | **0.014*** |  | 1.19 | 0.97-1.45 | 0.092 |
| D0 |  |  |  |  |  |  |  |  |  |  |  |  |
| LDH (U/L) | 203.8(56.8, 4364.5) | 1.16 | 0.82-1.66 | 0.400 |  | 0.97 | 0.70-1.35 | 0.862 |  | 1.50 | 1.08-2.08 | **0.016*** |
| Creatinine (mg/dL) | 0.3 (0.1, 0.7) | 0.96 | 0.49-1.92 | 0.916 |  | 0.75 | 0.41-1.35 | 0.330 |  | 1.44 | 0.53-2.60 | 0.399 |
| PLT (10^9^ cells/L) | 43.0 (2.0-289.0) | 0.95 | 0.69-1.31 | 0.766 |  | 1.00 | 0.76-1.31 | 0.983 |  | 0.78 | 0.56-1.10 | 0.162 |
| CRP (mg/L) | 7.9 (0.1-171.1) | 1.11 | 0.93-1.32 | 0.240 |  | 1.07 | 0.93-1.25 | 0.342 |  | 1.36 | 1.09-1.69 | **0.006*** |
| D7 |  |  |  |  |  |  |  |  |  |  |  |  |
| LDH (U/L) | 218.4 (84.8, 2664.0) | 1.52 | 0.96-2.42 | 0.075 |  | 1.17 | 0.69-1.98 | 0.567 |  | 1.91 | 1.14-3.20 | **0.014*** |
| Creatinine (mg/dL) | 0.3 (0.1, 1.3) | 1.31 | 0.71-2.43 | 0.392 |  | 0.96 | 0.55-1.66 | 0.870 |  | 1.60 | 0.56-4.59 | 0.384 |
| PLT (10^9^ cells/L) | 31.0 (6.0-99.0) | 0.77 | 0.57-1.16 | 0.215 |  | 0.78 | 0.55-1.10 | 0.151 |  | 0.46 | 0.26-0.81 | **0.007*** |
| CRP (mg/L) | 12.4 (0.0-177.6) | 1.28 | 1.04-1.56 | **0.018*** |  | 1.19 | 1.01-1.41 | **0.039*** |  | 1.42 | 1.11-1.82 | **0.005*** |
| D14 |  |  |  |  |  |  |  |  |  |  |  |  |
| LDH (U/L) | 345.4 (155.4, 9148.0) | 1.65 | 1.02-2.68 | **0.042*** |  | 1.52 | 0.98-2.36 | 0.062 |  | 2.02 | 0.96-4.27 | 0.065 |
| Creatinine (mg/dL) | 0.3 (0.1, 8.1) | 1.03 | 0.61-1.73 | 0.915 |  | 0.80 | 0.48-1.33 | 0.386 |  | 1.29 | 0.68-2.46 | 0.443 |
| PLT (10^9^ cells/L) | 43.0 (7.0-222.0) | 0.75 | 0.50-1.12 | 0.158 |  | 0.88 | 0.62-1.25 | 0.486 |  | 0.66 | 0.37-1.18 | 0.163 |
| CRP (mg/L) | 13.0 (0.1-124.9) | 1.21 | 1.00-1.47 | 0.054 |  | 1.19 | 1.01-1.41 | **0.033*** |  | 1.50 | 1.14-1.97 | **0.003*** |
| D30 |  |  |  |  |  |  |  |  |  |  |  |  |
| LDH (U/L) | 313.8 (153.3, 3110.9) | 1.67 | 0.83-3.36 | 0.152 |  | 1.42 | 0.78-2.59 | 0.255 |  | 1.33 | 0.25-7.13 | 0.742 |
| Creatinine (mg/dL) | 0.3 (0.1, 1.1) | 0.91 | 0.56-1.50 | 0.723 |  | 0.90 | 0.59-1.36 | 0.614 |  | 1.10 | 0.61-1.99 | 0.747 |
| PLT (10^9^ cells/L) | 111.0 (13.0-442.0) | 0.67 | 0.48-0.93 | **0.017*** |  | 0.64 | 0.43-0.97 | **0.034*** |  | 0.64 | 0.43-0.97 | **0.034*** |
| CRP (mg/L) | 0.7 (0.01-183.8) | 1.26 | 1.09-1.46 | **0.002*** |  | 1.30 | 1.15-1.48 | **< 0.001*** |  | 1.22 | 0.97-1.53 | 0.090 |

Abbreviation: OS, overall survival; RFS, relapse-free survival; NRM, non-relapse mortality. Log transformation of LDH, creatinine, PLT, and CRP values were applied to calculate the HR. ***** indicates statistically significant values.

**Supplementary Table 2** Patient Characteristics with High and Low D7-m-EASIX

|  | **D7-m-EASIX** | | | | | | |
| --- | --- | --- | --- | --- | --- | --- | --- |
|  | **Training cohort** | | ***P*-value** |  | **Validation cohort** | | ***P*-value** |
|  | **≤4.1 (N = 139)** | **>4.1 (N = 55)** |  |  | **≤4.1 (N = 85)** | **>4.1 (N = 24)** |  |
| Age (months) | 101.0 (9.0, 187.0) | 113.0 (19.0, 200.0) | **0.023** |  | 106.0 (9.0, 213.0) | 129.5 (10.0, 202.0) | 0.154 |
| Sex, n (%) |  |  | 0.819 |  |  |  | 0.386 |
| Male | 86 (61.9) | 35 (63.6) |  |  | 52 (61.2) | 17 (70.8) |  |
| Female | 53 (38.1) | 20 (36.4) |  |  | 33 (38.8) | 7 (29.2) |  |
| Disease, n (%) |  |  | 0.225 |  |  |  | 0.134 |
| ALL | 62 (44.6) | 19 (34.5) |  |  | 34 (40.0) | 15 (62.5) |  |
| AML | 70 (50.4) | 36 (65.4) |  |  | 48 (56.5) | 9 (37.5) |  |
| MDS | 7 (5.0) | 1 (1.8) |  |  | 3 (3.5) | 0 (0.0) |  |
| Status, n (%) |  |  | 0.317 |  |  |  | 0.070 |
| CR | 122 (87.8) | 51 (92.7) |  |  | 83 (97.6) | 21 (87.5) |  |
| Non-CR | 17 (12.2) | 4 (7.3) |  |  | 2 (2.4) | 3 (12.5) |  |
| MRD |  |  | 0.922 |  |  |  | 0.484 |
| Positive | 25 (18.0) | 9 (16.4) |  |  | 6 (7.1) | 3 (12.5) |  |
| Negative | 105 (75.5) | 43 (78.2) |  |  | 76 (89.4) | 21 (87.5) |  |
| NA | 9 (6.5) | 3 (5.5) |  |  | 3 (3.5) | 0 (0.0) |  |
| TBI |  |  | 0.534 |  |  |  | 0.099 |
| Yes | 17 (12.2) | 5 (9.1) |  |  | 15 (17.6) | 1 (4.2) |  |
| No | 122 (87.8) | 50 (90.9) |  |  | 70 (82.4) | 23 (95.8) |  |
| ABO matched |  |  | 0.149 |  |  |  | 0.799 |
| matched | 70 (50.4) | 34 (61.8) |  |  | 45 (52.9) | 12 (50.0) |  |
| mismatched | 69 (49.6) | 21 (38.2) |  |  | 40 (47.1) | 12 (50.0) |  |
| Donor-recipient sex match, n (%) |  |  | 0.124 |  |  |  | 0.410 |
| female to male | 16 (11.5) | 11 (20.0) |  |  | 6 (7.1) | 3 (12.5%) |  |
| others | 123 (88.5) | 44 (80.0) |  |  | 79 (92.9) | 21 (87.5) |  |
| Graft source, n (%) |  |  | 0.877 |  |  |  | **0.025*** |
| PB | 42 (30.2) | 16 (29.1) |  |  | 31 (36.5) | 3 (12.5) |  |
| PB+BM | 97 (69.8) | 39 (70.9) |  |  | 54 (63.5) | 21 (87.5) |  |
| HLA compatibility, n (%) |  |  | 0.773 |  |  |  | 0.426 |
| 5/10 | 89 (64.0) | 34 (61.8) |  |  | 49 (57.6) | 16 (66.7) |  |
| Others | 89 (64.0) | 21 (38.2) |  |  | 36 (42.4) | 8 (33.3) |  |
| GVHD prevention, n (%) |  |  | 0.656 |  |  |  | 1.000 |
| CSA + MMF + MTX | 129 (92.8) | 50 (90.9) |  |  | 83 (97.6) | 24 (100.0) |  |
| FK506 + MMF + MTX | 10 (7.2) | 5 (9.1%) |  |  | 2 (2.4) | 0 (0.0) |  |
| MNC (× 10^8^ /kg) | 7.0 (1.3, 21.7) | 7.3 (0.6, 12.2) | 0.779 |  | 6.6 (3.8-17.7) | 7.5 (4.0-11.5) | 0.233 |
| CD34+cell (× 10^6^ /kg) | 7.3 (1.7, 18.3) | 6.0 (1.4, 13.7) | **0.001*** |  | 7.4 (3.2-15.0) | 6.7 (3.7-13.5) | 0.432 |
| Engraftment time (days) |  |  |  |  |  |  |  |
| Neutrophil | 12.0 (9.0, 21.0) | 11.0 (9.0, 20.0) | 0.526 |  | 12.0 (9.0-21.0) | 11.0 (10.0-16.0) | 0.084 |
| Platelet | 11.0 (5.0, 40.0) | 11.0 (7.0, 24.0) | 0.096 |  | 11.0 (8.0-39.0) | 13.0 (7.0-61.0) | 0.058 |
| Abbreviations: AML, acute myeloid leukemia; ALL, acute lymphoblastic leukemia; MDS, myelodysplastic syndrome; CR, complete remission; MRD, minimal residual disease; TBI, total body irradiation; PB, Peripheral blood; BM, bone marrow; HLA, human leukocyte antigen; GVHD, graft versus host disease; CSA, cyclosporine A; FK506, tacrolimus; MMF, mycophenolate mofetil; MNC, mononuclear cells.  ***** represent statistical significance *p* < 0.05.   \| Cause of death \| **Training cohort** \| \|  \| **Validation cohort** \| \| \| --- \| --- \| --- \| --- \| --- \| --- \| \| Low D7-m-EASIX (N = 17) \| High D7-m-EASIX (N = 17) \|  \| Low D7-m-EASIX (N = 3) \| High D7-m-EASIX (N = 8) \| \| Original disease, n (%) \| 10 (58.8) \| 4 (23.5) \|  \| 1 (33.3) \| 3 (37.5) \| \| Infection, n (%) \| 3 (17.6) \| 6 (35.3) \|  \| 2 (66.7) \| 0 (0.0) \| \| GVHD \| 3 (17.6) \| 4 (23.5) \|  \| 0 (0.0) \| 2 (25.0) \| \| chemotherapy-related toxicity \| 1 (5.9) \| 0 (0.0) \|  \| 0 (0.0) \| 1 (12.5) \| \| TAM \| 0 (0.0) \| 2 (11.8) \|  \| 0 (0.0) \| 2 (25.0) \| \| Missing \| 0 (0.0) \| 1 (5.9) \|  \| 0 (0.0) \| 0 (0.0) \| \| Abbreviation: EASIX, Endothelial Activation and Stress Index; GVHD: graft-versus-host disease. TAM, transplant-associated microangiopathy. \| \| \| \| \| \|   **Supplementary Table 3** Reasons for deaths | | | | | | | |

| Variable | OS | | |  | RFS | | |  | NRM | | |
| --- | --- | --- | --- | --- | --- | --- | --- | --- | --- | --- | --- |
|  | HR | 95% CI | *P* value |  | HR | 95% CI | *P* value |  | HR | 95% CI | *P* value |
| Sex (male VS female) | 1.52 | 0.490-5.72 | 0.539 |  | 2.56 | 0.73-9.00 | 0.142 |  | 0.75 | 0.18-3.20 | 0.694 |
| Age | 1.01 | 0.99-1.02 | 0.358 |  | 1.01 | 1.00-1.02 | 0.329 |  | 1.00 | 0.99-1.02 | 0.760 |
| Disease (AML & MDS VS ALL) | 0.14 | 0.03-0.64 | **0.011*** |  | 0.35 | 0.12-0.96 | **0.042*** |  | 0.11 | 0.01-0.84 | **0.033*** |
| Disease status (non-CR VS CR) | 3.28 | 0.41-25.98 | 0.261 |  | 2.24 | 0.29-17.12 | 0.437 |  | 5.10 | 0.66-40.32 | 0.122 |
| MRD (positive VS negative) | 2.76 | 0.59-12.80 | 0.195 |  | 1.93 | 0.44-8.50 | 0.386 |  | 2.05 | 0.26-16.38 | 0.500 |
| TBI (yes VS no) | 1.27 | 0.27-5.88 | 0.762 |  | 2.11 | 0.68-6.56 | 0.199 |  | 0.94 | 0.12-7.67 | 0.956 |
| Blood-type match (mismatched vs matched) | 1.28 | 0.39-4.21 | 0.683 |  | 0.82 | 0.30-2.20 | 0.691 |  | 1.47 | 0.34-6.37 | 0.604 |
| Donor-recipient sex match (female to male vs others) | 3.00 | 0.65-13.94 | 0.161 |  | 1.99 | 0.45-8.77 | 0.364 |  | 2.07 | 0.25-16.88 | 0.496 |
| Graft source (PB+BM vs PB) | 2.15 | 0.46-9.94 | 0.329 |  | 2.12 | 0.60-7.44 | 0.241 |  | 1.15 | 0.23-5.84 | 0.866 |
| HLA compatibility (5/10 vs others) | 1.22 | 0.37-3.99 | 0.746 |  | 1.25 | 0.59-2.64 | 0.560 |  | 1.96 | 0.43-8.93 | 0.382 |
| Graft Dose |  |  |  |  |  |  |  |  |  |  |  |
| MNC | 1.05 | 0.83-1.33 | 0.691 |  | 1.08 | 0.90-1.30 | 0.390 |  | 1.05 | 0.90-1.21 | 0.555 |
| CD34 | 0.90 | 0.66-1.23 | 0.507 |  | 0.88 | 0.68-1.14 | 0.350 |  | 0.83 | 0.70-0.98 | **0.028*** |
| CMV (positive VS negative) | 4.78 | 1.74-13.12 | **0.002*** |  | 2.95 | 1.19-7.29 | **0.019*** |  | 2.38 | 0.48-11.80 | 0.288 |
| EBV (positive VS negative) | 0.49 | 0.11-2.26 | 0.358 |  | 0.49 | 0.14-1.72 | 0.265 |  | 0.37 | 0.05-2.95 | 0.347 |
| BSI (positive VS negative) | 2.13 | 0.62-7.29 | 0.229 |  | 2.21 | 0.80-6.10 | 0.124 |  | 5.09 | 1.18-21.86 | **0.029*** |
| D7-m-EASIX (high VS low) | 6.84 | 2.00-23.38 | **0.002*** |  | 3.07 | 1.14-8.25 | **0.026*** |  | 9.30 | 1.82-47.47 | **0.007*** |
| Abbreviation: OS, overall survival; RFS, relapse-free survival; NRM, non-relapse mortality; AML, acute myeloid leukemia; ALL, acute lymphoblastic leukemia;  MDS, myelodysplastic syndrome; CR, complete remission; MRD, minimal residual disease; TBI, total body irradiation; PB, Peripheral blood; BM, bone marrow;  HLA, human leukocyte antigen; MNC, mononuclear cells; CMV, Cytomegalovirus; EBV, Epstein-Barr virus; BSI, bloodstream infections.  *****represent statistical significance p ≤ 0.10. | | | | | | | | | | | |

**Supplementary Table 4** Univariate analysis of risk factors for main outcomes in validation cohort

| Variable | OS | | |  | RFS | | |  | NRM | | |
| --- | --- | --- | --- | --- | --- | --- | --- | --- | --- | --- | --- |
|  | HR | 95% CI | *P* value |  | HR | 95% CI | *P* value |  | HR | 95% CI | *P* value |
| Disease (AML & MDS VS ALL) | 0.27 | 0.05-1.42 | 0.123 |  | 0.50 | 0.17-1.50 | 0.217 |  | 0.15 | 0.02-1.14 | 0.067 |
| CD34 |  |  |  |  |  |  |  |  | 0.99 | 0.80-1.23 | 0.940 |
| BSI (positive VS negative) |  |  |  |  |  |  |  |  | 4.59 | 0.95-22.24 | 0.058 |
| CMV (positive VS negative) | 5.02 | 1.80-13.99 | **0.002*** |  | 3.12 | 1.16-7.70 | **0.014*** |  |  |  |  |
| D7-m-EASIX (high VS low) | 6.56 | 1.76-24.42 | **0.005*** |  | 3.20 | 1.12-9.15 | **0.030*** |  | 5.32 | 1.08-26.24 | **0.040*** |
| Abbreviation: OS, overall survival; RFS, relapse-free survival; NRM, non-relapse mortality; AML, acute myeloid leukemia; ALL, acute lymphoblastic leukemia;  MDS, myelodysplastic syndrome; BSI, bloodstream infections; CMV, Cytomegalovirus.  ***** represent statistical significance p < 0.05 | | | | | | | | | | | |

**Supplementary Table 5** Multivariate analysis of risk factors for main outcomes in validation cohort

| Variable | Univariate analysis | | |  | Multivariate analysis | | |
| --- | --- | --- | --- | --- | --- | --- | --- |
|  | HR | 95% CI | *P* value |  | HR | 95% CI | *P* value |
| Sex (male VS female) | 1.18 | 0.56-2.47 | 0.669 |  |  |  |  |
| Age | 0.99 | 0.99-1.01 | 0.781 |  |  |  |  |
| Disease (AML & MDS VS ALL) | 0.83 | 0.41-1.70 | 0.613 |  |  |  |  |
| Disease status (non-CR VS CR) | 0.68 | 0.10-4.46 | 0.687 |  |  |  |  |
| MRD (positive VS negative) | 1.28 | 0.40-3.96 | 0.693 |  |  |  |  |
| TBI (positive VS negative) | 0.93 | 0.33-2.65 | 0.893 |  |  |  |  |
| Blood-type match (mismatched vs matched) | 0.60 | 0.29-1.26 | 0.177 |  |  |  |  |
| Donor-recipient sex match (female to male vs others) | 0.88 | 0.19-4.08 | 0.867 |  |  |  |  |
| Graft source (PB+BM vs PB) | 1.26 | 0.57-2.79 | 0.568 |  |  |  |  |
| HLA compatibility (5/10 vs others) | 1.22 | 0.60-2.52 | 0.582 |  |  |  |  |
| Graft Dose |  |  |  |  |  |  |  |
| MNC | 0.97 | 0.84-1.13 | 0.734 |  |  |  |  |
| CD34 | 0.96 | 0.83-1.11 | 0.541 |  |  |  |  |
| CMV (positive VS negative) | 2.45 | 1.16-5.20 | **0.019*** |  | 2.68 | 1.17-6.12 | **0.020*** |
| EBV (positive VS negative) | 2.02 | 0.72-5.63 | 0.181 |  |  |  |  |
| BSI (positive VS negative) | 2.51 | 1.16-5.45 | **0.020*** |  | 3.13 | 1.35-7.25 | **0.008*** |
| D7-m-EASIX (high VS low) | 2.96 | 1.44-6.07 | **0.003*** |  | 2.57 | 1.28-5.19 | **0.008*** |
| Abbreviation: GVHD, graft-versus-host disease. AML, acute myeloid leukemia; ALL, acute lymphoblastic leukemia;MDS, myelodysplastic syndrome; CR, complete remission; MRD, minimal residual disease; TBI, total body irradiation; PB, Peripheral blood; BM, bone marrow; HLA, human leukocyte antigen; MNC, mononuclear cells; CMV, Cytomegalovirus; EBV, Epstein-Barr virus; BSI, bloodstream infections.  ***** represent statistical significance p ≤ 0.10 in univariate analysis and p < 0.05 in multivariate analysis. | | | | | | | |

**Supplementary Table 6** Univariate analysis and multivariate analysis of II-IV aGVHD in validation cohort

Landscape of Gene Mutations and Fusion Transcripts in AM Samples. (A) training cohort; (B)validation cohort

A

**B**
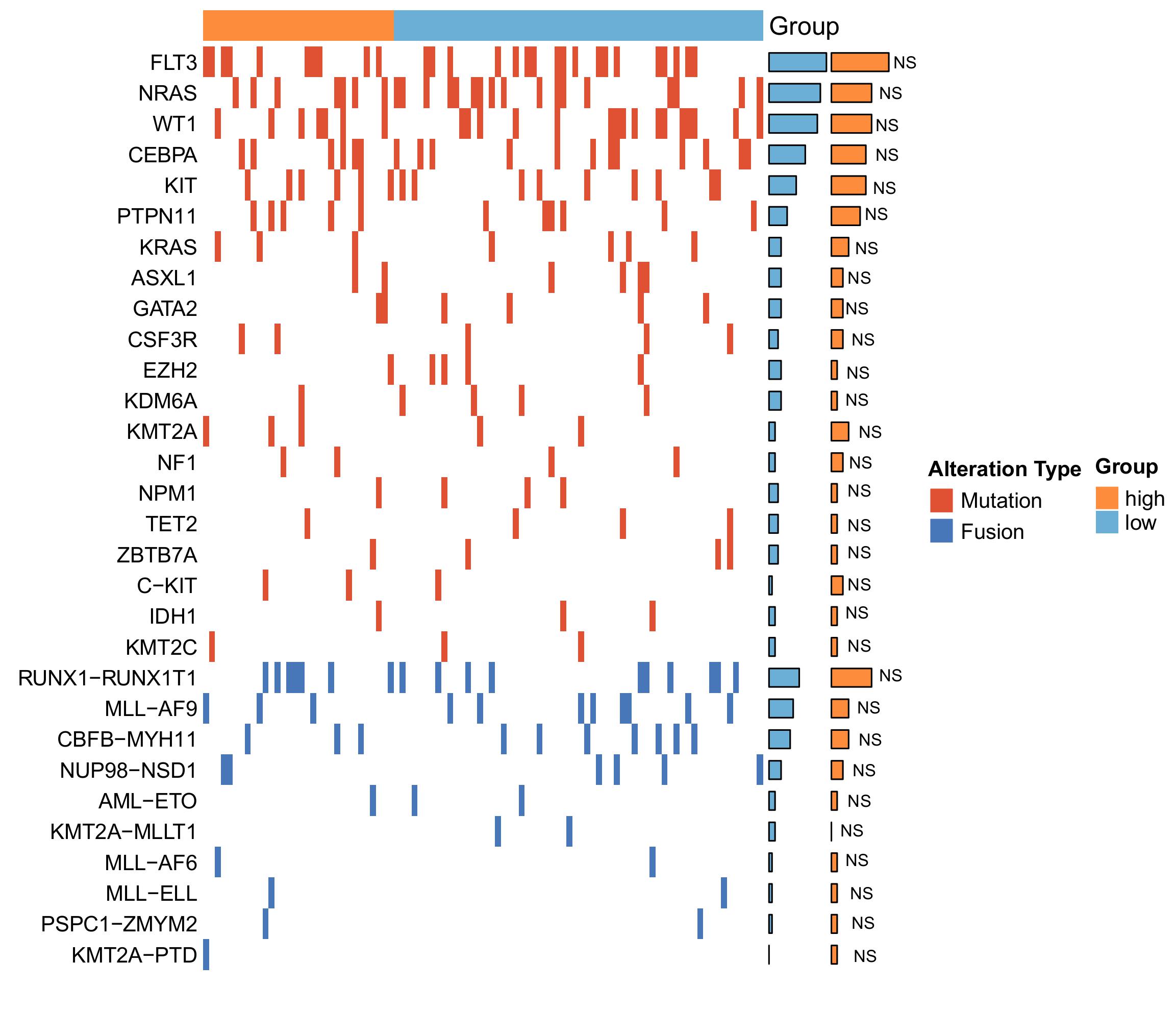
**
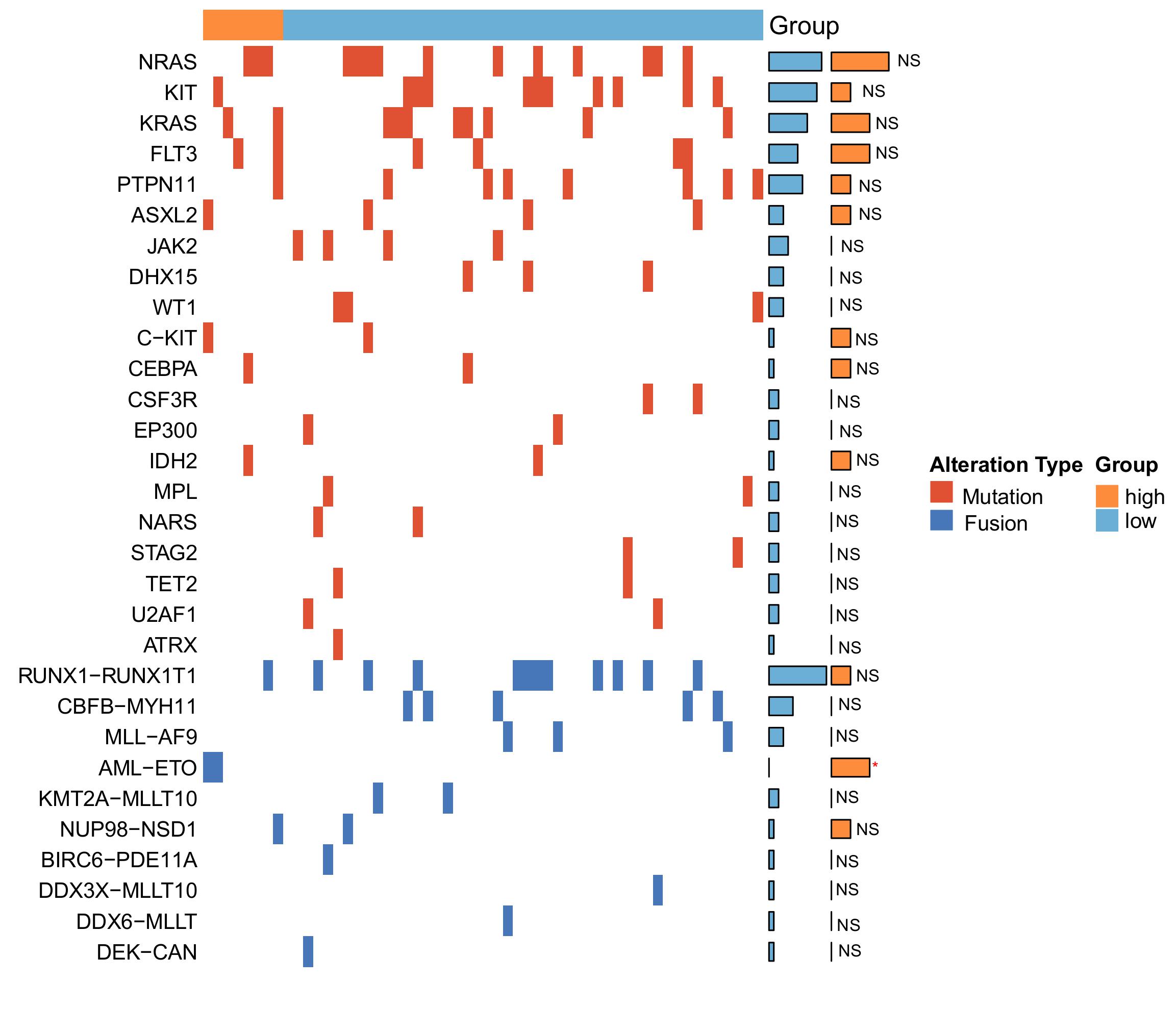
**

**Supplementary Fig 2** Time‑dependent hazard ratio of D7‑m‑EASIX for OS and RFS

**A**

**
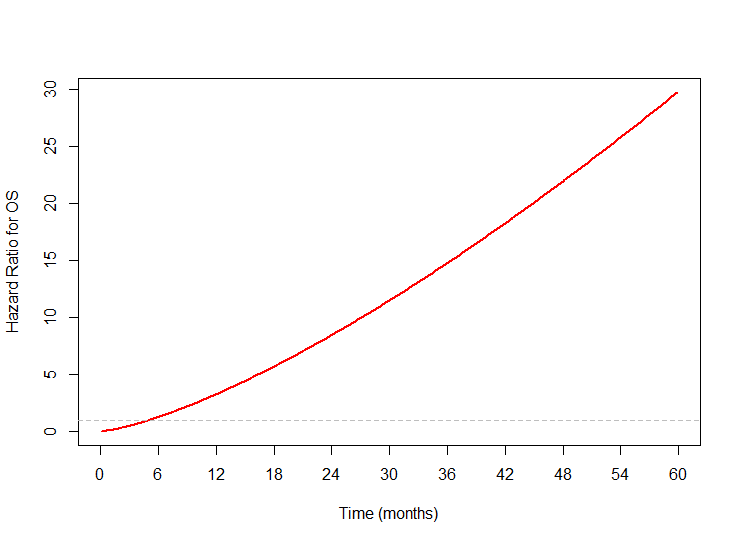
**

**B**

**
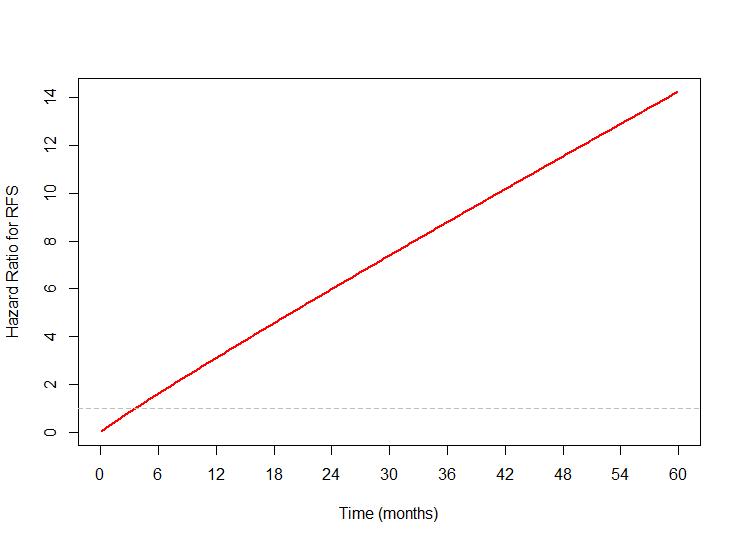
**

**Supplementary Fig 3** Overall survival and Relapse-free survival according to D7-m-EASIX score in validation cohort. (a) overall survival; (b) relapse-free survival.

**a**


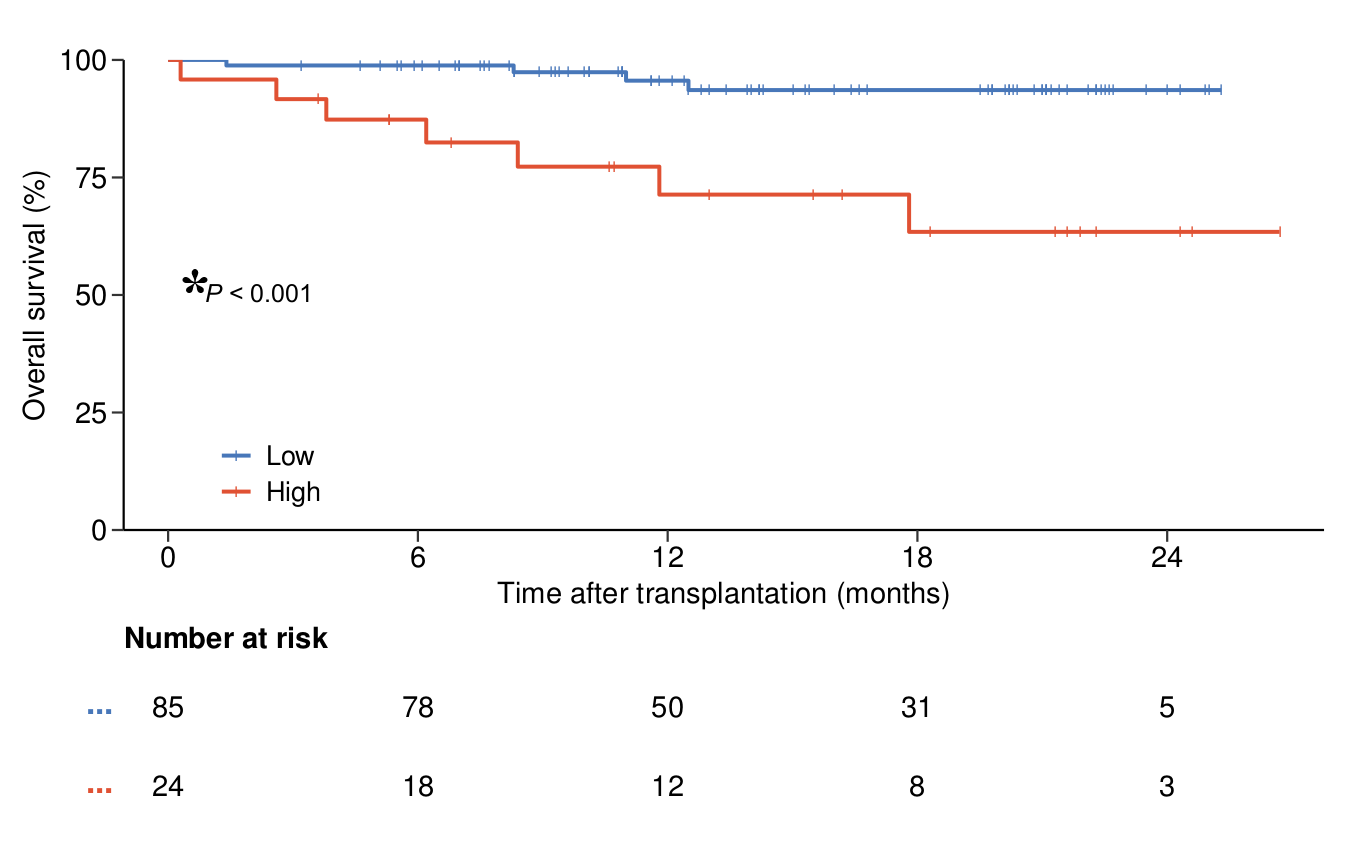


**b**
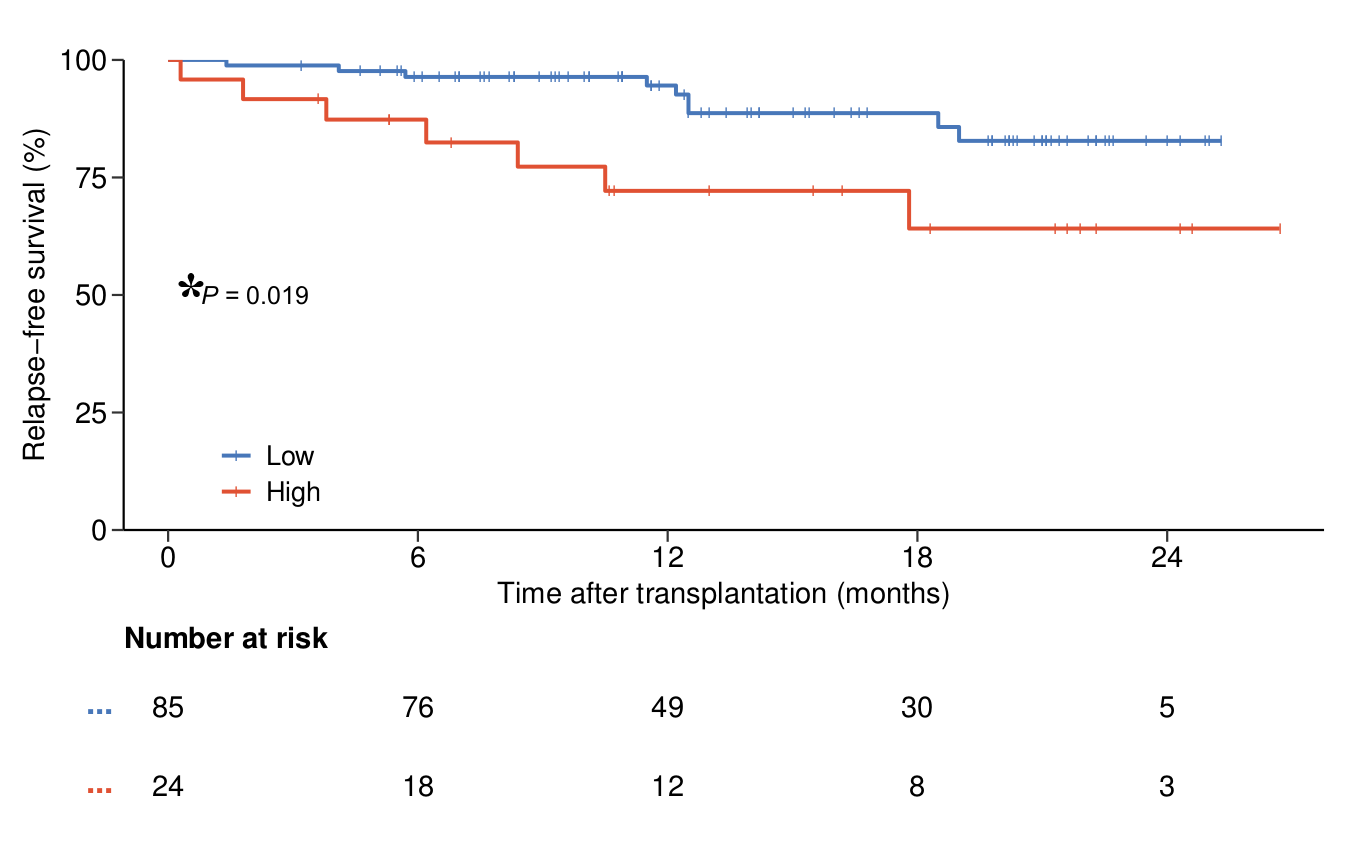


**Supplementary Fig 4** The cumulative incidences of relapse and non-relapse morality according to D7-m-EASIX score in validation cohort. (a) relapse; (b) non-relapse morality.

**a**
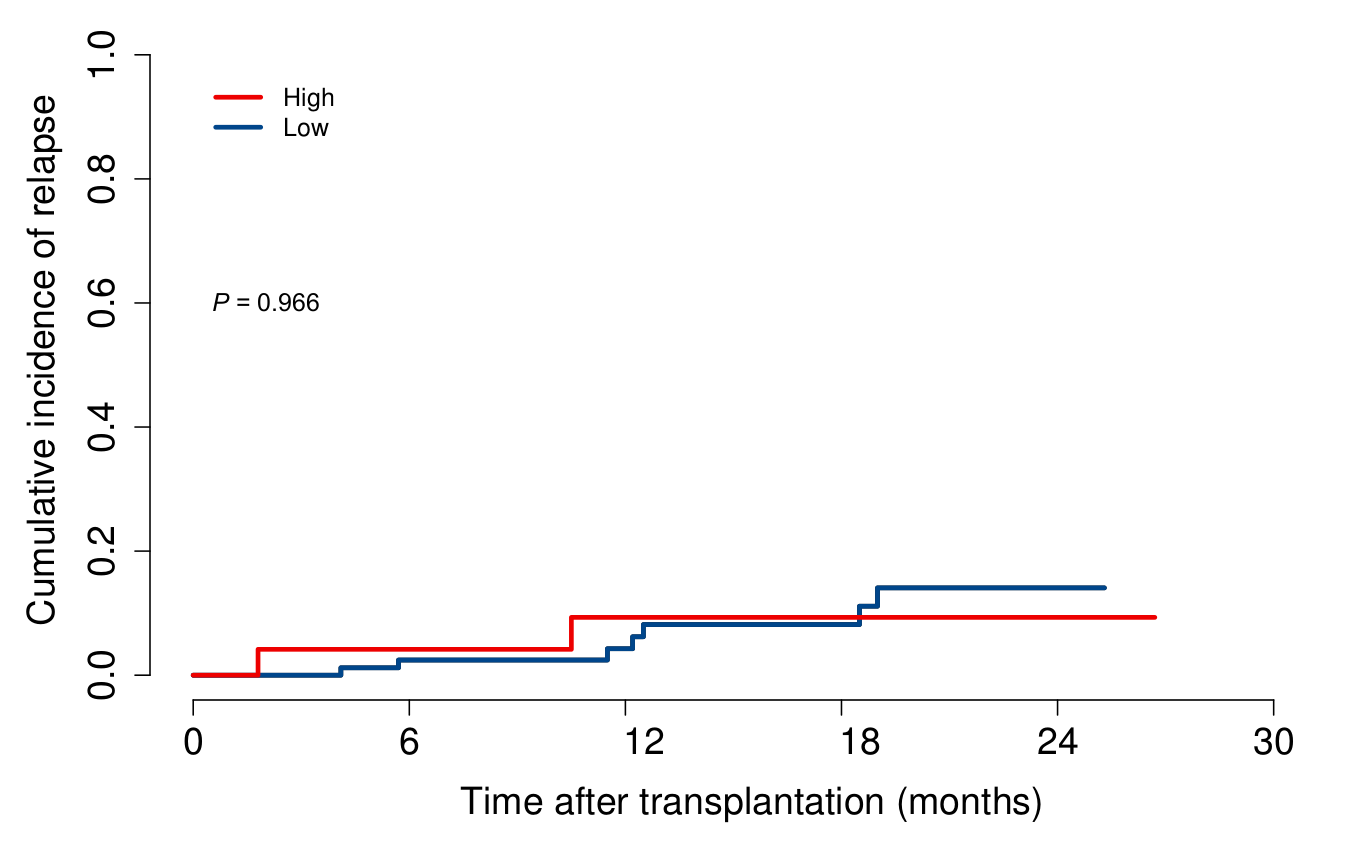


**b**
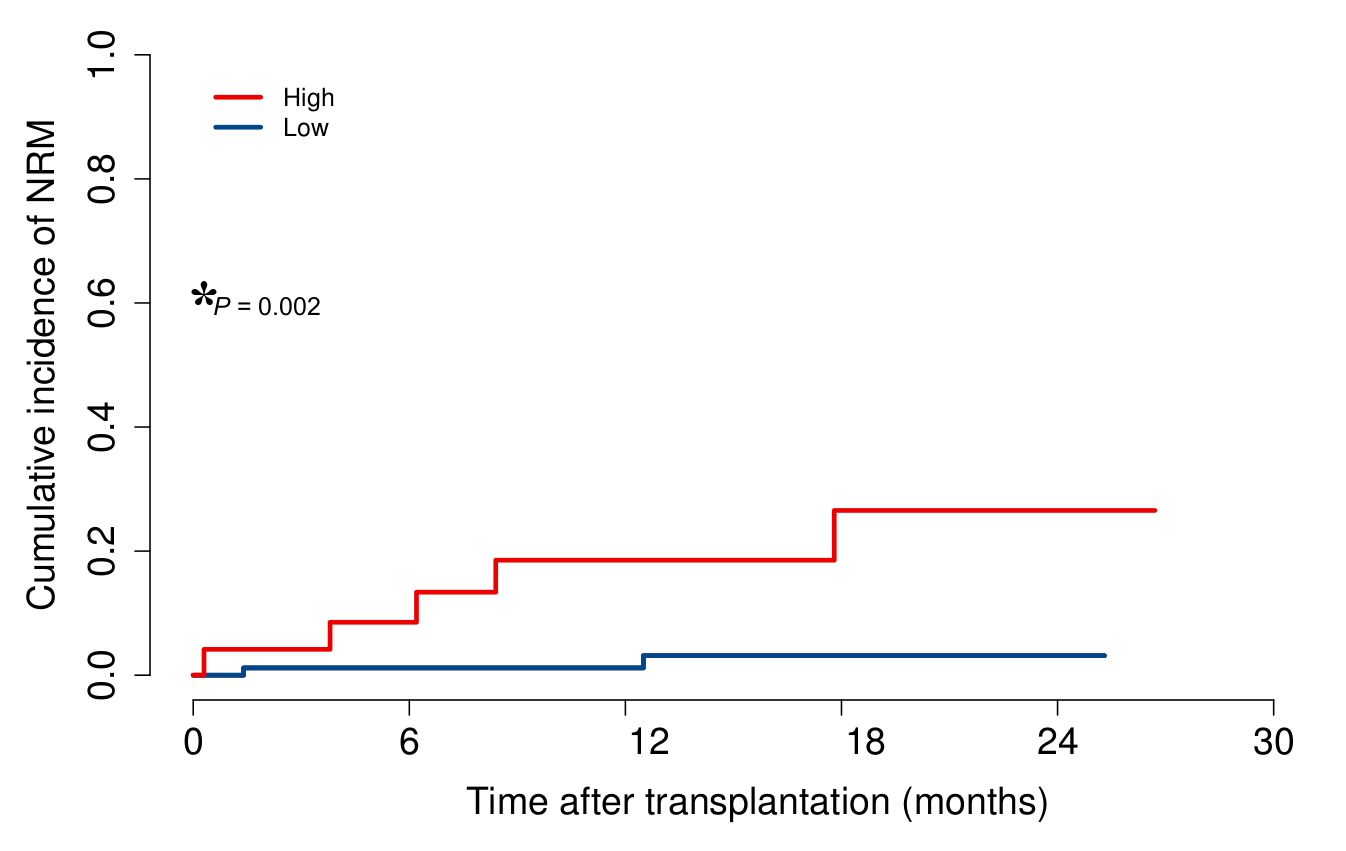


**Supplementary Fig 5** The impact of D7-m-EASIX score on GVHD in validation cohort. (a) cumulative incidence of II-IV aGVHD; (b) cumulative incidence of III-IV aGVHD; (c) cumulative incidence of cGVHD.

**a b**


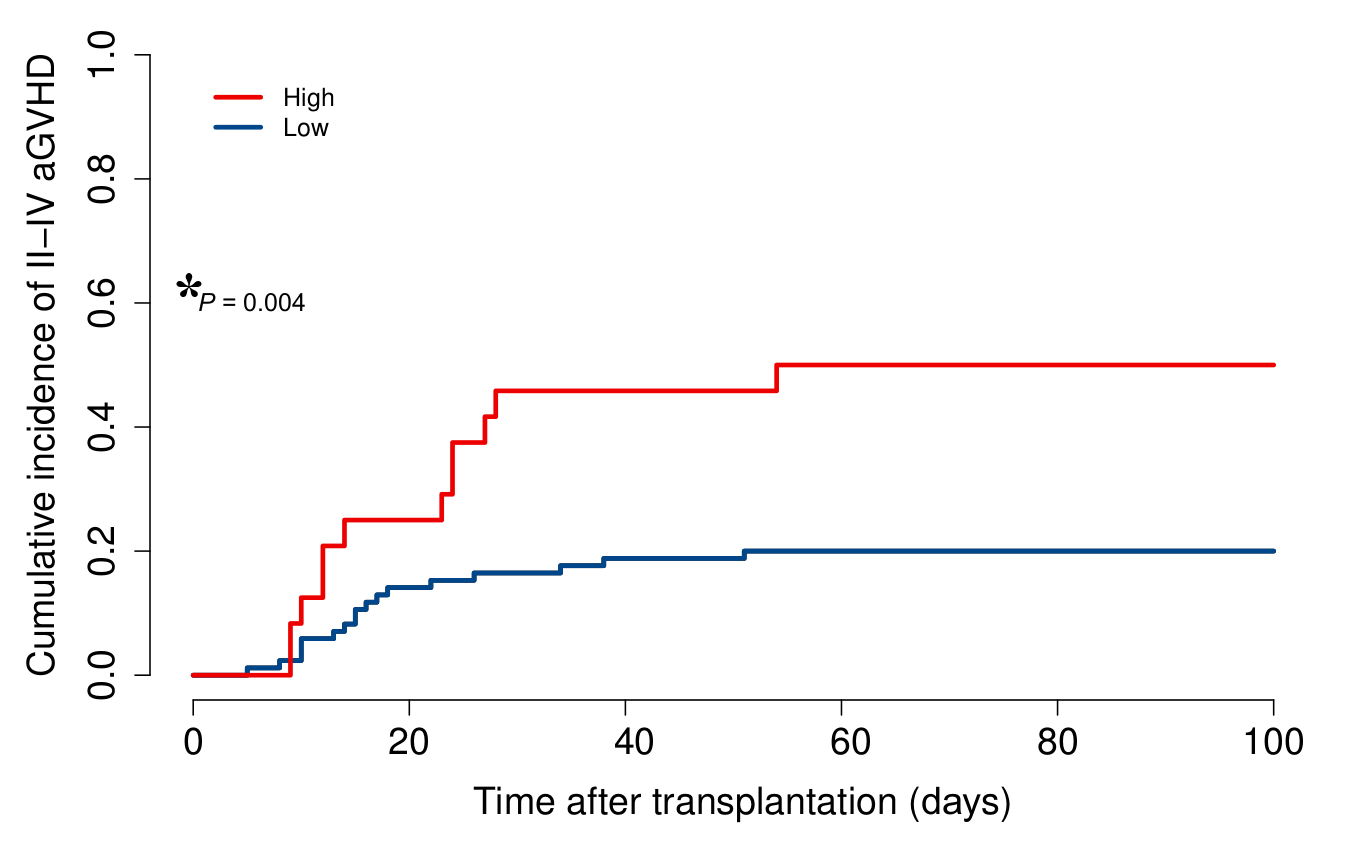

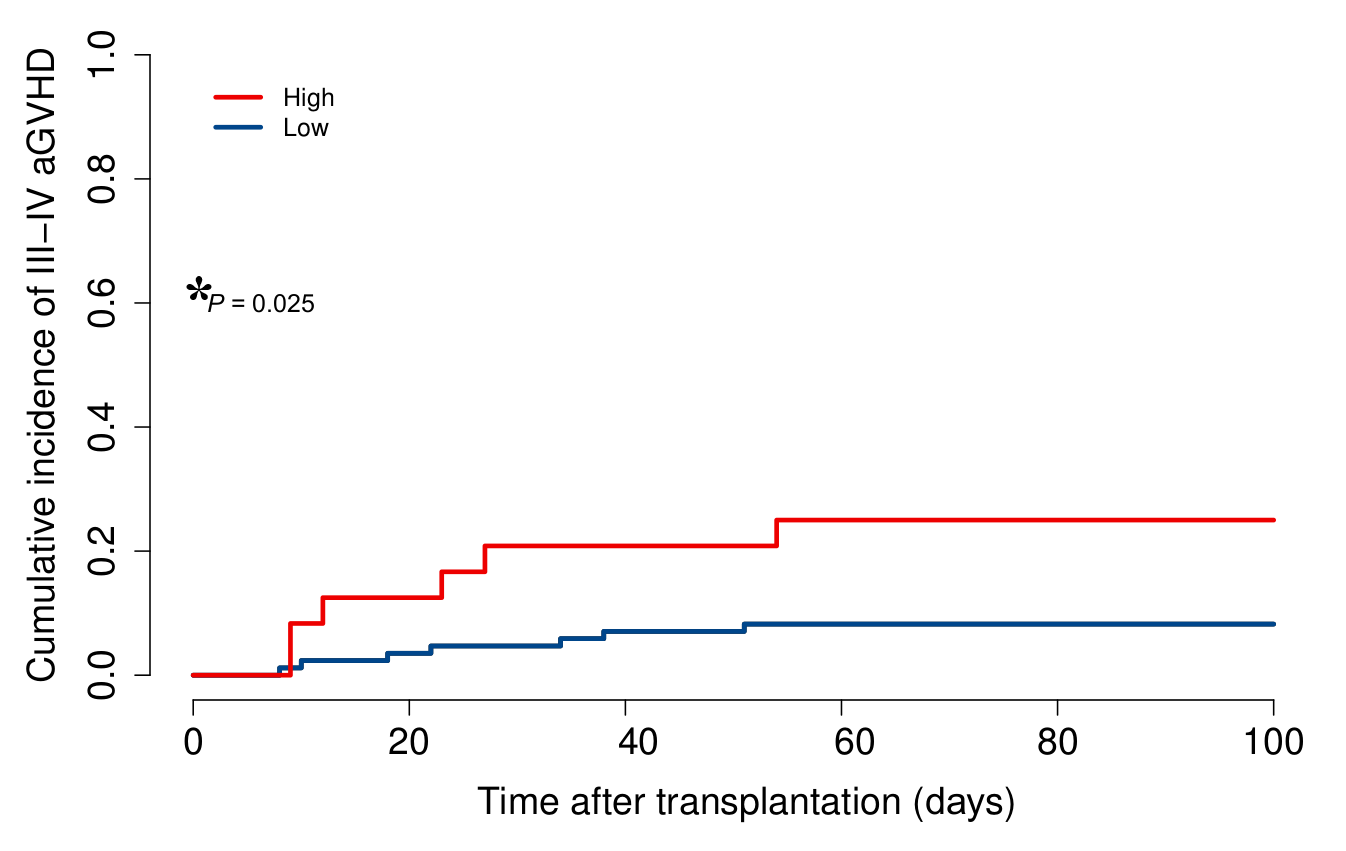


**c**


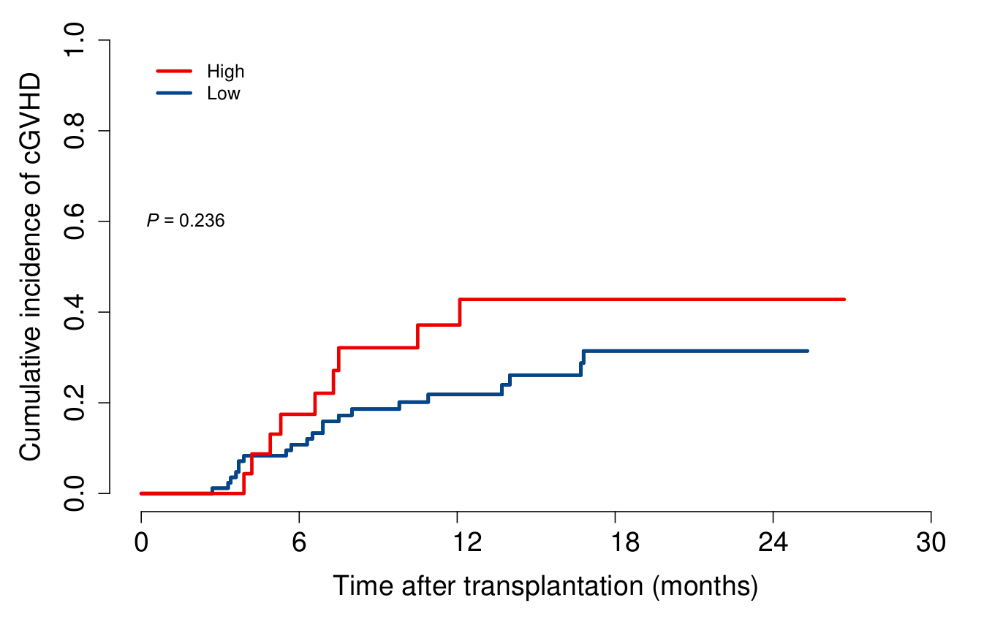

Supplement: Supplementary file 1 [file DataSheet1.docx]
